# Semi-Empirical Force-Field Model for the Ti_1−x_Al_x_N  (0 ≤ x ≤ 1) System

**DOI:** 10.3390/ma12020215

**Published:** 2019-01-10

**Authors:** G. A. Almyras, D. G. Sangiovanni, K. Sarakinos

**Affiliations:** 1 Nanoscale Engineering Division, Department of Physics, Chemistry, and Biology, Linköping University, SE 581 83 Linköping, Sweden; george.almyras@gmail.com; 2 Atomistic Modelling and Simulation, ICAMS, Ruhr-Universität Bochum, D-44801 Bochum, Germany; davide.sangiovanni@liu.se; 3 Theoretical Physics Division, Department of Physics, Chemistry, and Biology, Linköping University, SE 581 83 Linköping, Sweden

**Keywords:** titanium-aluminum nitride, Ti-Al-N, MD simulations, molecular dynamics, interatomic potential, MEAM, force-field model, spinodal decomposition, phase stability

## Abstract

We present a modified embedded atom method (MEAM) semi-empirical force-field model for the Ti_1−x_Al_x_N (0 ≤ x ≤ 1) alloy system. The MEAM parameters, determined via an adaptive simulated-annealing (ASA) minimization scheme, optimize the model’s predictions with respect to 0 K equilibrium volumes, elastic constants, cohesive energies, enthalpies of mixing, and point-defect formation energies, for a set of ≈40 elemental, binary, and ternary Ti-Al-N structures and configurations. Subsequently, the reliability of the model is thoroughly verified against known finite-temperature thermodynamic and kinetic properties of key binary Ti-N and Al-N phases, as well as properties of Ti_1−x_Al_x_N (0 < x < 1) alloys. The successful outcome of the validation underscores the transferability of our model, opening the way for large-scale molecular dynamics simulations of, e.g., phase evolution, interfacial processes, and mechanical response in Ti-Al-N-based alloys, superlattices, and nanostructures.

## 1. Introduction

Ti_1−x_Al_x_N (0 ≤ x ≤ 1) alloys are widely used for wear and oxidation protection of metal cutting and forming tools, aerospace components, and automotive parts [1,2]. The parent binary phases B1-TiN (space group Fm–3m) and B4-AlN (space group P6_3_mc) are immiscible at room temperature and at thermodynamic equilibrium [3]. However, far-from-equilibrium conditions, which prevail during vapor-based thin-film deposition [4], enable synthesis of metastable B1-Ti_1−x_Al_x_N solid solutions for x ≤ 0.7 [5]. These metastable alloys undergo, in the temperature range ≈1000 to ≈1200 K, spinodal decomposition into strained isostructural Al-rich and Ti-rich B1-Ti_1−x_Al_x_N domains, which in turn, results in an age-hardened material with superior high-temperature mechanical and oxidation performance [6].

Over the past two decades, numerous theoretical and experimental studies [6,7,8,9,10,11,12,13] have focused on the mechanical properties and chemical stability, kinetics and energetics of structure formation, and phase stability of Ti_1−x_Al_x_N (0 ≤ x ≤ 1) alloys, during vapor-based thin-film synthesis and high-temperature operation. Despite the knowledge generated from these investigations, atomic-scale processes that drive phase transformations (including spinodal decomposition) and elastic/plastic response in B1-Ti_1−x_Al_x_N alloys (0 ≤ x ≤ 1) are poorly understood. This is because the time and length scales at which these processes occur often lie beyond the temporal and spatial resolution of experimental techniques, while their study using first-principle theoretical methods is currently computationally unfeasible.

Classical molecular dynamics (CMD) simulations complement first-principle methods for studying atomic-scale processes in a multitude of materials science problems. In CMD, the force field between particles in an atomic assembly is efficiently determined via semi-empirical mathematical models, also referred to as classical interatomic potentials [14,15,16,17,18,19], which reduce the many-body interactions of electrons and nuclei to effective interactions between atom cores. CMD can describe phenomena necessitating the use of up to 10^6^–10^7^ atoms during times reaching 10^−6^–10^−5^ s, which cannot be achieved using first-principle methods. Examples of such phenomena include crystal growth, mass transport, plastic deformation, interfacial reactions, phase separation, and structural transformations. 

The reliability of CMD simulations depends critically on the choice of the type (i.e., the mathematical formalism) of the interatomic potential and the specific values of its parameters. These choices become exceptionally challenging when the aim is to describe solids that are characterized by the coexistence of competing phases, and a complex mixture of covalent, ionic, and metallic bonds, as, e.g., the Ti_1−x_Al_x_N alloys [20]. The second-neighbor modified embedded atom method (MEAM) interatomic potential [21] is an empirical model that has reliably reproduced physical attributes of elemental phases, as well as compounds and alloys in crystalline [22,23,24,25,26,27,28,29] and amorphous [30] form, including B1-TiN [23,31], fcc-Al [26,32], hcp- [33] and bcc-Ti [34], and N_2_ dimers [35,36]. However, the MEAM models available in the literature are not suitable for describing kinetic and thermodynamic properties of the Ti_1−x_Al_x_N system over the entire composition range (0 ≤ x ≤ 1), and for different phases and configurations. Hence, a complete re-parametrization of all elemental, pair, and triplet Ti-Al-N interactions is required for developing a MEAM Ti_1−x_Al_x_N (0 ≤ x ≤ 1) interatomic potential.

In this study, we embark on the above-mentioned task by employing an adaptive simulated-annealing (ASA) methodology to efficiently probe the multi-dimensional MEAM parameter space and select parameter values that optimize the potential’s predictions with respect to a large set of reference Ti-Al-N material properties. These include 0 K equilibrium volumes, cohesive energies, elastic constants, point-defect formation energies, and mixing enthalpies for ≈40 Ti-Al-N phases and structures. Subsequently, we validate the transferability of the potential for structures and physical attributes not explicitly included in the ASA optimization procedure by studying finite-temperature kinetic and thermodynamic properties of key Ti-N and Al-N phases, as well as properties of Ti_1−x_Al_x_N (0 < x < 1) alloys. We find that our potential reproduces the lattice and elastic constants, phonon-spectra, defect migration energies, phase diagrams, and solid–solid phase transitions induced by temperature and/or pressure changes within the Ti_1−x_Al_x_N (0 ≤ x ≤ 1) system remarkably well. This opens the way for large-scale molecular dynamics simulations of Ti-Al-N phase transformations and elastic/plastic responses, which may provide critical insights for designing Ti-Al-N-based superlattices and nanostructures with superior thermal stability and mechanical performance.

The paper is organized as follows. Section 2 contains a description of the methodology used for the parametrization and validation of the MEAM potential. Section 3 presents the MEAM parameters and the results of the optimization procedure, followed by results and discussion of the potential validation in Section 4. Finally, the overall results are summarized in Section 5, which also discusses the relevance of our study for large-scale simulations within the Ti_1−x_Al_x_N system and beyond.

## 2. Potential Parametrization and Validation Methodology

### 2.1. Potential Parametrization Methodology

The MEAM interatomic model potential for compounds and alloys is well-documented in the literature [21,23,24], and hence, it is not explained in detail here. The specific mathematical formalism employed in this work is elaborated in Section S1 of the Supplemental Materials.

An effective and complete description of all pair and triplet interactions within the Ti-Al-N system requires the selection of values for ≈10^2^ MEAM parameters (see Table S1 in Supplementary Materials). We address this formidable challenge by using a stochastic algorithm based on an adaptive simulated annealing (ASA) scheme [37], which is explained in detail in Section S2 and illustrated in Figure S3 in Supplementary Materials. This algorithm allows us to determine parameter values that optimize the potential’s predictions relative to experimentally- and theoretically-determined physical properties of phases and configurations of Al, Ti, and N and their binary and ternary combinations, as explained later in the present section. This is a necessary, yet not sufficient, condition for developing a MEAM potential which is fully transferable among different compositions with varying x values (0 ≤ x ≤ 1) and metal-sublattice atomic arrangements within the Ti_1−x_Al_x_N alloy system. To reduce the size of the multi-dimensional parameter-space that needs to be scanned during the ASA optimization procedure, we constrained the range of key MEAM parameters, i.e., interatomic distance *r_e_*, cohesive energy *E_c_*, bulk modulus *B* (*E_c_*, *B*, and *r_e_* determine the MEAM parameter *α*), and attractive *d_+_* and repulsive *d_–_* interaction, to be close to values that best fit density functional theory (DFT) total energies versus equilibrium volumes (i.e., equation of states), for all reference elemental and binary phases. More details and results on the above-mentioned DFT calculations can be found in Section S1 and in Figures S1 and S2 in Supplementary Materials.

The phases used in the parametrization procedure are divided into two groups: (i) Metallic systems, i.e., hexagonal (hcp) α-Ti (space group P6_3_/mmc), cubic (bcc) β-Ti (space group Im–3m), hexagonal ω-Ti (space group P6/mmm), cubic (fcc) γ-Al (space group Fm–3m), cubic γ-Al_98_Ti_2_ random alloys, cubic L1_2_ Al_3_Ti (space group Pm–3m), tetragonal D0_22_ Al_3_Ti (space group I4/mmm), tetragonal L1_0_ AlTi (space group P4/mmm), hexagonal Ti_3_Al (space group P6_3_/mmc), and hexagonal α-Ti_98_Al_2_ random alloys. (ii) Nitrogen-based systems, i.e., N_2_ dimer, linear N_3_ trimer, triangular N_3_ molecule, cubic B1-TiN (rocksalt structure), tetragonal rutile ε-Ti_2_N (space group P4_2_/mnm), B1-AlN, B3-AlN (cubic zincblende structure, space group F–43m), B4-AlN (hexagonal wurtzite structure), and nineteen ternary random solid solutions of B1-Ti_1−x_Al_x_N with equidistant compositions with x in the range 0.05 to 0.95. 

For the phases listed above, we used ASA to determine sets of MEAM parameters that optimize the potential’s predictions for the following 0 K physical properties: (i) cohesive energies (*E_c_*); (ii) lattice parameters (a, c), and elastic constants (bulk moduli *B* and *C_ij_*) for all phases; (iii) mixing enthalpies (*ΔH_mix_*) for B1-Ti_1−x_Al_x_N random solid solutions calculated with respect to the energies of B1-TiN and B1-AlN parent binary phases (*ΔH_mix_* = *E_c,B1-Ti1−xAlxN_* − (1–x)·*E_c,B1-TiN_* − x·*E_c,B1-AlN_*); and (iv) point-defect (vacancies V and interstitials I) formation energies *E^f^_V/I_* for B1-TiN and B1-AlN. 

Bulk moduli and lattice parameters were determined using least-square fitting of the total energy versus equilibrium volume curves, while the elastic constants *C_ij_* were calculated using least-square fitting of energy versus strain curves for structures at their equilibrium volumes. *E^f^_V/I_* values were computed with respect to the chemical potential *μ* of Ti, Al, and N in hcp-Ti, fcc-Al, and N_2_ molecules, respectively. Specifically, *E^f^_V/I_* = *E_D_* ± *μ* – *E*_0_, where *E_D_* is the energy of a crystal containing one point-defect, *E*_0_ is the energy of the defect-free crystal, while the sign preceding *μ* is positive for vacancies and negative for interstitials. All physical properties mentioned above were determined via MEAM conjugate–gradient energy minimization calculations at 0 K using lattices consisting of 250 to 450 atoms. Exception to the latter are the B1-Ti_1−x_Al_x_N systems, where all properties were evaluated by averaging over the results obtained for 10 different random cation–sublattice configurations using supercells formed by 1000 atoms. For reference, we also calculated N and Ti vacancy formation energies in B1-TiN with respect to the chemical potential of isolated Ti and N atoms by means of both MEAM and DFT. The latter was done using the VASP code [38], the Perdew–Burke–Ernzerhof (PBE) approximation [39] for the exchange and correlation energy, the projector augmented wave (PAW) method to describe electron-core interactions [40], and supercells formed of 215 atoms + 1 vacancy. The total energy of the relaxed TiN system was evaluated to an accuracy of 10^–5^ eV/supercell, using 3 × 3 × 3 k-point grids and 400 eV cutoff-energy for planewaves. The energy of isolated N and Ti atoms was computed accounting for spin relaxation.

The quality of each MEAM parameter set was further assessed (during the ASA optimization) by evaluating the potential’s performance with respect to structural stability, melting points (*T_m_*), and phase transitions induced in AlN upon changing temperature *T* and/or pressure *P*. This was accomplished by means of 5 to 30 ps long CMD simulations at finite *T* and *P* values for cell sizes of ≈1000 atoms. Structural stability was tested by verifying that the simulated systems exhibit no unphysical behavior during 30 ps and that total energies are conserved during microcanonical *NVE* sampling at temperatures of ≈0.5 *T_m_*. Melting points were rapidly estimated as the temperature values for which the derivative of equilibrium volumes versus *T* becomes sharply discontinuous by performing 5 ps CMD isobaric–isothermal *NPT* simulations at different temperatures near experimental *T_m_* values. The relative stability of competing phases was assessed by calculating the free energies of such phases on a grid of *T* and/or *P* values via thermodynamic integration (TI) [41]. The reference energy for TI is the free energy of an Einstein crystal, i.e., a system of non-interacting harmonic oscillators with frequencies determined from the mean square displacements (MSD) of the material system under consideration [42]. Using the assessment process described above, along with the ASA optimization scheme, we defined the best set of MEAM parameters, which is validated as explained in Section 2.2.

### 2.2. Potential Validation Methodology

In order to explore the transferability of the parameters determined using the procedure described in Section 2.1, we studied the following properties and dynamic processes that are not explicitly included in the ASA optimization scheme: (i) lattice thermal expansion of B4-AlN and B1- Ti_1−x_Al_x_N (0 ≤ x ≤ 1); (ii) finite-temperature phonon spectra of B1-TiN, B1-AlN, and B4-AlN; (iii) nitrogen- and metal-vacancy migration energies for B1-TiN, B1-AlN, and B4-AlN, as well as diffusion energetics of Al interstitials in B1-TiN; (iv) lattice and elastic constants of sub-stoichiometric B1-TiN_y_ (0.7 ≤ y ≤ 1), B2-TiN (space group Pm–3m), body-centered tetragonal (bct) Ti_2_N (space group I4_1_/amd); (v) free energies of B1-AlN and B4-AlN and dynamics of B4- to B1-AlN phase transformation; and (vi) B1-Ti_1−x_Al_x_N alloy mixing free energies with respect to B1-TiN and B1-AlN. 

The first step for studying the effect of temperature on the lattice parameter is to determine the temperature-dependent equilibrium volumes of different phases. This was done by performing CMD simulations employing *NPT* sampling of the phase space, using the Langevin thermostat coupled to the Parrinello–Rahman barostat [43], with settings suggested in Ref. [41]. The supercells used for these simulations are typically formed of ≈500 atoms. Equilibrium volumes and lattice parameters versus *T* (0 < *T* ≤ 3300 K) curves were then fitted to second-order polynomials to obtain the lattice thermal expansion coefficient.

Finite-temperature phonon spectra and free energies (including vibrational entropies) were obtained using post-simulation processing of CMD forces and atomic displacements via the temperature-dependent effective-potential (TDEP) method [44,45,46], using both second-order and third-order force constants. The TDEP method, which allows for the evaluation of the dynamic matrix directly at a temperature of interest, has been successfully applied for understanding the vibrational effects on the dynamic stabilization of crystal lattices [47] and solid–solid phase transitions [48]. For TDEP simulations in this work, we used canonical *NVT* sampling at equilibrium volumes previously determined via *NPT* sampling at a given temperature and pressure, and we controlled the temperature via the Nose–Hoover thermostat. Moreover, we compared CMD phonon-spectra to those obtained via TDEP applied to density-functional ab initio molecular dynamics (AIMD) [49] simulation results. AIMD/TDEP data are available in the literature for B1-AlN and B4-AlN [50]. For B1-TiN, we carried out additional AIMD simulations (VASP, PAW, PBE, 300 eV cutoff energy, and Γ-point sampling) of 10-ps duration on supercells formed of 512 atoms at temperatures of 300 and 1200 K. For calculating the free energy of mixing for B1-Ti_1−x_Al_x_N random solid solutions, we imposed a symmetric force constant matrix in the framework of TDEP. This was implemented by using reference ideal B1 primitive cells with the metal sublattice occupied using a single elemental species *Q* of mass *M_Q_* = (1–x)·*M_Ti_* + x·*M_Al_*, as proposed in Ref. [51]. The configurational entropy *S* = –*k_B_* [x·ln(x) + (1–x)·ln(1–x)] per formula unit of B1-Ti_1−x_Al_x_N alloys was evaluated via the mean-field theory approximation. Binodal and spinodal compositions x were determined as a function of temperature. Binodal points, which separate stable from metastable alloy regions in the composition space, were obtained from the tangents to the B1-Ti_1−x_Al_x_N mixing free energy curves [52]. Spinodal points, which separate metastable from unstable B1-Ti_1−x_Al_x_N regions, were determined from the change in sign in the second derivative of alloy mixing free energies versus x [52].

Vacancy and interstitial migration energies, as well as minimum-energy paths, were obtained via static MEAM nudged-elastic band (NEB) [53,54] calculations. The elastic constants of B1-TiN_y_ (0.7 ≤ y ≤ 1) were determined by averaging over the values calculated for ≈100 different configurations (supercells containing 512 lattice sites) using the scheme described in Ref. [55]. The dynamics of B4- to B1-AlN transformation was studied via *NPT* CMD simulations at 300 K using a timestep of 0.1 fs. First, a supercell containing ≈20000 B4 AlN atoms was thermally equilibrated. Then, a pressure ramp with an average rate ≈7 GPa·ps^–1^ was used during CMD/*NPT* simulations for a total duration of ≈0.2 ns. A video of the AlN transformation, produced with the Visual Molecular Dynamics [56] software, can be found in the Supplemental Material.

All 0 K calculations and finite-temperature CMD simulations, described in Section 2.1 and Section 2.2, were performed using the LAMMPS package [57] (software updated to version of August 10, 2015). Timesteps of 1 fs (or less) were used for the integration of the equations of motion during CMD and AIMD runs. All CMD data presented in Section 3 and Section 4 were obtained using our MEAM Ti_1−x_Al_x_N (0 ≤ x ≤ 1) potential.

## 3. Potential Parametrization Results

The MEAM parameters, as determined by the procedure described in Section 2.1, are listed in Table S1 in Supplemental Material and are also available online [58,59]. In addition, we provide scripts that allow a prompt start of CMD simulations for interested readers (see Supplemental Material). The predictions of the potential with respect to the properties of the metallic reference phases (see Section 2.1) are provided in the Supplemental Material file (Section S3, Table S3, Figure S4–S7), where the cohesive energy and the interatomic distances for N_2_ and N_3_ molecules can also be found (Table S2) (see Supplemental Materials).

The predicted cohesive energies, lattice parameters, and elastic constants for B1-TiN, ε-Ti_2_N, B1-AlN, B3-AlN, and B4-AlN along with their respective experimental (in brackets) and DFT (in parentheses) reference literature values [36,60,61,62,63,64,65,66,67,68,69,70,71,72,73,74,75,76,77,78,79,80,81,82,83,84,85,86,87,88,89] are listed in Table 1. MEAM results for lattice parameters are in excellent agreement with DFT and experimental data. In addition, elastic properties calculated using our MEAM potential are, in general, in very good agreement with the reference values. Furthermore, our potential reproduces the established trend qualitatively with respect to the energetics (i.e., *E_c_*) of AlN polymorph structures; B4-AlN is more stable than B3-AlN, which is, in turn, more stable relative to B1-AlN. From a quantitative point of view, the cohesive energy difference *E_c,B3-AlN_* – *E_c,B4-AlN_* of 30 meV/atom calculated from MEAM is within the range of DFT values (≈20–40 meV/atom), while the MEAM *E_c,B1-AlN_* – *E_c,B4-AlN_* value of 68 meV/atom is lower than DFT predictions (≈150–200 meV/atom). Moreover, we find that the MEAM parameters yield an asymmetric B1-Ti_1−x_Al_x_N *ΔH_mix_* versus x curve that exhibits a downward concavity and a maximum of ≈230 meV/f.u at x = 0.6. This is within the range of DFT predictions by Alling et al. [3,90,91,92], Wang et al. [93], and Mayrhofer et al. [94], which found *ΔH_mix_* maxima in the interval ≈210–280 meV/f.u. for x ≈ 0.62.

*E^f^_V_* values for N and Ti vacancies (N_V_ and Ti_V_, respectively), obtained using a N atom in a N_2_ molecule and of a Ti atom in hcp-Ti lattice as reference chemical potentials, are *E^f^_NV_* = 3.53 eV and *E^f^_TiV_* = 4.76 eV. The corresponding DFT-based values, found in the literature, lie in the ranges 2.41–2.53 eV (N_V_) [95,96] and 2.86–3.11 eV (Ti_V_) [96,97]. The discrepancy between MEAM and DFT results becomes less than 10% when using the chemical potential of isolated N and Ti for computing *E^f^_V_*. In this case, MEAM yields *E^f^_NV_* = 8.41 eV and *E^f^_TiV_* = 9.81 eV, while the corresponding DFT values are *E^f^_NV_* = 7.52 eV and *E^f^_TiV_* = 9.71 eV.

The vacancy formation energies in AlN, estimated using MEAM calculations with respect to the energies of a N atom in a N_2_ dimer and of Al atom in fcc Al, are *E^f^_NV_* = 2.15 eV and *E^f^_AlV_* = 5.25 eV for B1-AlN, and *E^f^_NV_* = 3.50 eV and *E^f^_AlV_* = 6.66 eV for B4-AlN. DFT results [98,99] are in the range between –3 and 4 eV for N_V_ and –2 and 10 eV for Al_V_, i.e., in the same order of magnitude with our MEAM values. It is important to note that the formation energy of point defects in semiconductors is largely determined by their respective charge state. Since the MEAM formalism does not account for charge states, a strict quantitative comparison between classical and ab initio data for vacancy formation energies in AlN is not meaningful.

**Figure 1:**
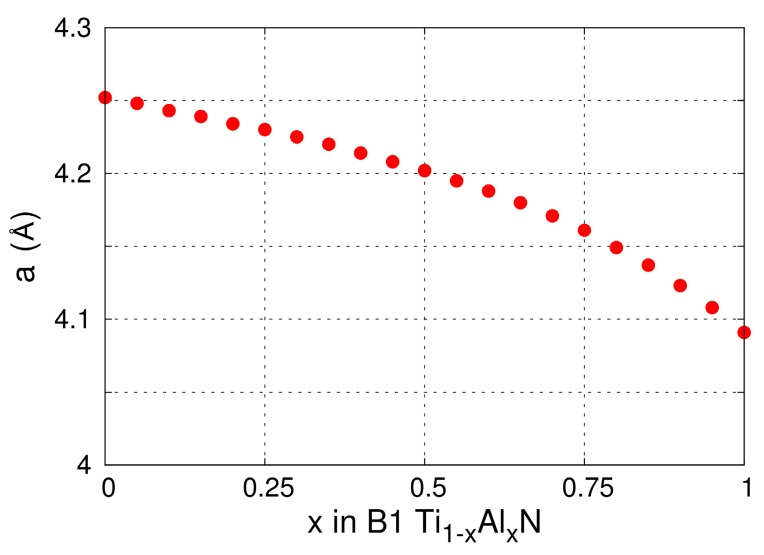
MEAM-predicted lattice parameter a of B1-Ti_1−x_Al_x_N (0 ≤ x ≤ 1) as function of x.

**Figure 2:**
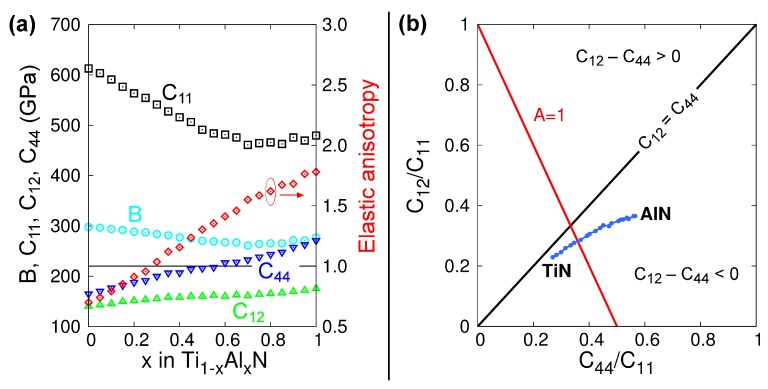
(**a**) Elastic constants *B* (cyan circles), *C_11_* (black squares), *C_12_* (green triangles), and *C_44_* (blue inversed triangles), as well as Zener’s elastic anisotropy *A* = 2·*C_44_*/(*C_11_*–*C_12_*) (red diamonds), as a function of x within the Ti_1−x_Al_x_N system. The red circle and arrow are used to indicate that the elastic anisotropy data point refers to the right-axis scale. (**b**) Blackman’s diagram constructed using data from (a). The 45° solid black line represents the zero Cauchy pressure, *C_12_* = *C_44_*. The red solid line represents the conditions for elastic isotropy *A* = 1.

The lattice parameters of B1-Ti_1−x_Al_x_N solid solutions versus x calculated from MEAM at 0 K (see Figure 1) are in excellent agreement with DFT (see figure 2 in Ref. [100] and figure 6 in Ref. [92]) and experimental (see figure 6 in Ref. [92]) results. The elastic constants and Zener’s elastic anisotropy factor *A* = 2·*C_44_*/(*C_11_–C_12_*) obtained from MEAM calculations for B1-Ti_1−x_Al_x_N are plotted as a function of x in Figure 2a. The bulk moduli *B* and *C_11_* elastic constantly decrease, while *C_44_*, *C_12_*, and the factor *A* increase monotonically with increasing x. This evolution is in excellent agreement with DFT data [101]. Moreover, our potential yields the same as in DFT calculations [101], stoichiometry (x = 0.28) at which B1-Ti_1−x_Al_x_N solid solutions are elastically isotropic (*A* = 1). The latter is a strong indication that our MEAM parameters reproduce, in a reliable fashion, interatomic forces and energetics of Ti-Al-N systems.

Figure 2b presents a plot of *C_12_*/*C_11_* versus C_44_/*C_11_* ratios calculated from our MEAM potential. This representation is known as Blackman’s diagram [102] and entails information on the elastic anisotropy and bonding characteristics of the crystal. The black solid line in Figure 2b represents the condition *C_12_* = *C_44_*. The regions above (below) the *C_12_* = *C_44_* line indicate positive (negative) Cauchy’s pressures *C_12_* − *C_44_*. Based on phenomenological observations, Pettifor suggested that the Cauchy pressure can be used to assess the bonding character in cubic systems: positive values indicate metallic bonds, while negative values indicate more directional and covalent-like bonds [103]. We see that, for B1-Ti_1−x_Al_x_N alloys, *C_12_* − *C_44_* become progressively more negative for increasing AlN contents (blue curve in Figure 2b). This is due to the increasing directionality of the bonds caused by the presence of Al, and it is in agreement with DFT results [101].

The melting points *T_m_* of all investigated elementary systems, estimated from CMD simulations, are in reasonable agreement with their reference literature values (see Supplemental Material). In the case of AlN, we estimated a *T_m_* value of ≈3200 K. To our knowledge, the melting point of AlN has not been experimentally determined, and previous ab initio predictions indicate that AlN melts at approximately 3000 K [60]. For B1-TiN, our MEAM parameters yield a *T_m_* of approximately 6000 K, which is considerably higher than the experimental value (≈3250 K) [104]. It should be emphasized that CMD simulations using *NPT* sampling in defect-free crystals may lead to overestimations of melting points relative to values predicted using CMD modelling of solid–liquid interfaces at equilibrium [105]. It is reasonable to expect that such overestimations are greater for TiN versus AlN since the absence of polymorphs competing with the B1-TiN at atmospheric pressure reduces the possibility of nucleating heterogeneous sites that can facilitate melting.

## 4. Potential Validation Results

Classical potentials often lack transferability, i.e., they fail to accurately calculate interatomic forces in chemical environments different than those used in parameter fitting schemes. This means that the ability of the MEAM parameters listed in the Supplemental Material to reproduce 0 K physical properties of reference phases is a necessary, but not sufficient, condition for describing the dynamic evolution and thermodynamic properties of Ti_1−x_Al_x_N (0 ≤ x ≤ 1) alloys. Hence, in this section we present a thorough validation of the proposed set of Ti-Al-N MEAM parameters focusing on finite-temperature properties, which are difficult to implement within the ASA scheme. We study lattice thermal expansion and dynamics, defect migration energetics, and structural stability and transformations in key binary Ti-N and Al-N phases. These properties, in combination with studies of phase energetics in a multitude of Ti_1−x_Al_x_N (0 < x < 1) alloy compositions, provide a solid foundation for assessing the robustness and the reliability of the potential beyond the phases and configurations used in the ASA procedure.

### 4.1. Lattice Thermal Expansion

The lattice thermal expansion is an important feature in crystalline solids, as it reflects changes in interatomic forces as a function of temperature. Determination of thermal expansion using CMD simulations is a simple and rapid test for unravelling unphysical lattice instabilities and structural transformations, which may indicate poor potential parameter quality and/or insufficient transferability of the potential formalism.

**Figure 3:**
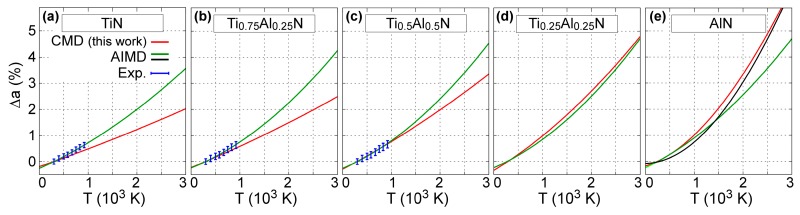
Variation of B1-Ti_1−x_Al_x_N lattice parameter a as a function of temperature *T* {∆a(*T*) = [a(*T*) – a(300 K)]/a(300 K)} for (**a**) x = 0, (**b**) x = 0.25, (**c**) x = 0.5, (**d**) x = 0.75, and (**e**) x = 1. AIMD (solid green and black curves) and experimental (blue dots) results are adapted from figure 1 in Ref. [71]. AIMD curves (green lines) shown in the present figure were obtained by fitting the data in the temperature range 0 to 2000 K of Ref. [71] with a second order polynomial. The AIMD curve (solid black line) in (**e**) was obtained using the data presented for B1-AlN equilibrium volume versus *T* in figure 2a in Ref. [50].

The temperature-induced variation in B1-Ti_1−x_Al_x_N (x = 0, 0.25, 0.5, 0.75, and 1) lattice parameters obtained via CMD simulations is represented in Figure 3. The CMD results (red solid lines in Figure 3a–e) are in good agreement with previous experimental (blue dots in Figure 3a–c) and AIMD simulation data (green solid lines in Figure 3a–e) and black solid line in Figure 3e) [50,71]. 

Closer inspection of Figure 3a shows that our MEAM parameters yield a room-temperature B1-TiN lattice constant of 4.26 Å, which is slightly larger (<1%) than the corresponding experimental value of 4.24 Å [106]. In addition, static (i.e., 0 K) calculations using our MEAM potential show that a_B1-TiN_ (0 K) = 4.252 Å (see Table 1), which is within the range of 0 K DFT calculations (4.188–4.256 Å) based on different electronic exchange/correlation approximations [36]. The relative increase in lattice parameter values calculated for *T* = 2000 K via CMD simulations is equal to 1.2%, which is comparable to AIMD predictions (2.0%) from Ref. [59] (see solid green line in Figure 3a). The mean linear thermal expansion coefficient of B1-TiN is thus calculated to monotonically increase from 6.7 × 10^–6^ K^–1^ at 300 K to 8.1 × 10^–6^ K^–1^ at 3000 K. The MEAM estimates are slightly lower than the corresponding experimental values, which lay in the range (7–10) × 10^–6^ K^–1^ [104,107,108,109]. Even though our potential underestimates the thermal expansion of B1-TiN, the discrepancy between MEAM and experimental a_B1-TiN_ is within ≈1% from room temperature up to 3000 K.

Figure 3 also shows that, according to CMD simulations, the variation ∆a_B1-TiAlN_ becomes more pronounced with increasing AlN content x. This is consistent with the results of DFT calculations based on the quasi-harmonic approximation, AIMD simulations [59], and synchrotron X-ray diffraction [109]. For instance, at *T* = 2000 K, ∆a_B1-TiAlN_(*T*) values determined using CMD are found to increase from 1.2 to 3.3% for x increasing from 0 (B1-TiN) to 1 (B1-AlN). For reference, AIMD simulation results [71] showed that ∆a_B1-TiAlN_ (2000 K) increases from 2.0 to 2.5% for the same x range.

Our CMD estimation of the B1-AlN thermal expansion closely matches recent AIMD results (figure 2a in Ref. [50]) obtained for temperatures between 0 and 3000 K (compare black vs. red curves Figure 3e). It should be noted that accurate experimental determination of the B1-AlN thermal expansion is difficult due to the metastable nature of this phase. To our knowledge, the only experimental data available [110], obtained for temperatures between 300 and 450 K, are scattered between 5 and 10 × 10^–6^ K^–1^. For the same temperature range, MEAM results, Figure 3e, yield a thermal expansion coefficient of ≈13 × 10^–6^ K^–1^.

**Figure 4:**
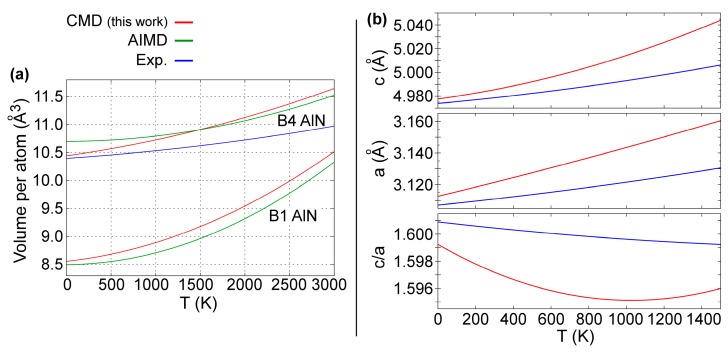
(**a**) Temperature dependence of B4-AlN and B1-AlN equilibrium volumes as predicted using CMD and AIMD simulations [50] and experiments [86]. The experimental curve (blue) was obtained from the a and c/a ratio variation versus T determined in Ref. [86]. (**b**) Temperature dependence of a, c, and c/a ratio of B4-AlN lattice parameters as predicted using CMD (red solid line) and measured using X-ray powder diffractometry (blue solid line) in the temperature range 300 to 1400 K from Ref. [86].

The temperature variation of B4-AlN equilibrium volumes between 0 and 3000 K is presented in Figure 4a. There, the B1-AlN data from Figure 3e are plotted again for the sake of clarity. Reproducing the temperature-dependence in equilibrium volumes and lattice parameters of hexagonal wurtzite crystals is particularly challenging due to anisotropic (in-plane vs. out-of-plane) structural properties and interatomic forces [86]. This notwithstanding, our CMD predictions (red solid lines) are in excellent agreement with both experimental (blue solid lines) [86] and AIMD (green solid lines) [50] results. Moreover, both CMD and AIMD curves presented in Figure 4a indicate that B4-AlN has a smaller thermal expansion than B1-AlN, which is consistent with synchrotron X-ray diffraction results from Ref. [109]. 

The B4-AlN a and c lattice parameters, as well as the c/a ratio are plotted versus *T* in Figure 4b. The results from our CMD simulations (red solid lines) are within 1% deviation from experimental values (blue solid lines) obtained via x-ray powder diffractometry measurements between 300 and 1400 K [86]. In addition, c/a versus *T* CMD data are also in agreement with experimental data in Figure 4b.

### 4.2. Lattice Dynamics

In order to ensure reliability of our MEAM model for large-scale CMD simulations, it is also necessary to test the way in which it reproduces vibrational properties of Ti-Al-N crystals. The study of lattice dynamics using the TDEP enables us to calculate phonon dispersions and phonon densities of states of Ti-Al-N systems directly at finite temperatures. This is useful to, e.g., verify the dynamic stability of crystal structures of interest and assess the effect of vibrational entropy on finite-temperature material properties. 

**Figure 5:**
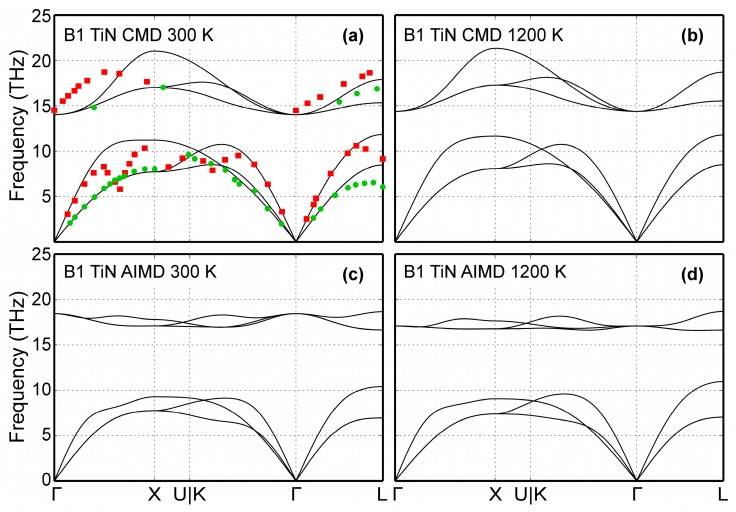
B1-TiN phonon dispersion curves calculated via: (**a**) CMD for 300 K, (**b**) CMD for 1200 K, (**c**) AIMD for 300 K, and (**d**) AIMD for 1200 K. Experimental data (green dots for transversal and red squares for longitudinal modes) obtained using neutron scattering measurements on B1-TiN_0.98_ bulk samples from Ref. [111] are included for comparison.

The B1-TiN phonon dispersion curves are presented in Figure 5 for two different temperatures (300 and 1200 K). CMD results are shown in Figure 5a,b, while phonon spectra obtained using AIMD simulations are plotted in Figure 5c,d. For comparison, Figure 5a includes experimental data obtained at room temperature for B1-TiN_0.98_ compounds. Vibrational frequencies measured via neutron scattering [111] are shown as green circles (transversal modes) and red squares (longitudinal modes). The 300 K CMD dispersion curves (Figure 5a) are in good qualitative agreement with both experimental data (Figure 5a) and AIMD results of Figure 5c. Moreover, our CMD simulations show that an increase of temperature from 300 (Figure 5a) to 1200 K (Figure 5b) does not significantly affect the vibrational frequencies in B1-TiN, in qualitative agreement with the corresponding AIMD data in Figure 5c,d. On a quantitative level, we observe that CMD predicted optical phonon bandwidths of ≈6 THz (Figure 5a,b), which is larger than the corresponding values from experiments (≈4 THz, Figure 5a) [100] and AIMD (≈2 THz, Figure 5c,d). We also found that CMD simulations cannot not reproduce the phonon softening observed in experimental and AIMD data (Kohn anomalies on acoustic modes at L zone boundaries and on the Γ→X and Γ→K paths), since the screening function employed in the MEAM formalism [21] removes long-range interactions. 

**Figure 6:**
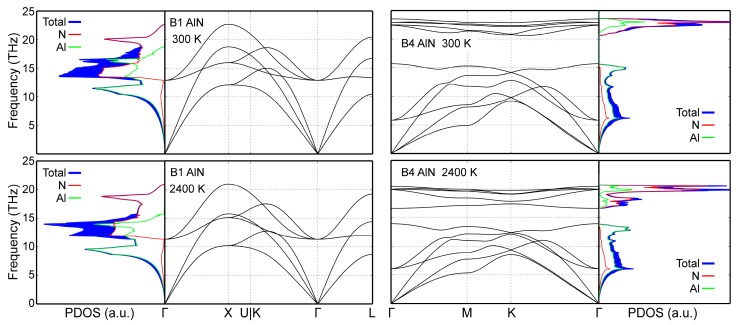
B1- and B4-AlN CMD phonon spectra and phonon densities of states (PDOS) calculated at 300 and 2400 K.

Phonon density of states (PDOS) data in Figure 6 demonstrate that our MEAM potential correctly yield dynamically stable (all phonon frequency values are positive, i.e., real) B1- and B4-AlN structures at both room and elevated temperatures (2400 K). The same held for B3-AlN at 300 K (data not shown). Moreover, total PDOS curves and site-projected populations of acoustic and optical states obtained from our simulations are quantitatively close to those obtained via AIMD at both 300 and 2400 K [50]. The dynamic stability evidenced in Figure 6, combined with the accurate reproduction of 0 K cohesive energies in Section 3, shows that our MEAM potential provides a complete description of all AlN polymorphs with a single parameter set.

CMD acoustic phonon frequencies calculated for B1- and B4-AlN at 300 and 2400 K are, overall, in good agreement with AIMD results (compare Figure 6 with figure 4 in Ref. [50]). It is important to note that MEAM cannot reproduce the splitting of degenerate longitudinal and transverse optical phonons [112], which is inherent to polarizable materials such as AlN (see, for example, optical frequencies near the Γ point in Figure 6 and those obtained ab initio in Ref. [50]). This is due to the fact that the MEAM formalism does not explicitly include Coulomb interactions. 

### 4.3. Point-Defect Migration Energies

The set of MEAM parameters selected via the ASA procedure was also tested with respect to the energetics of point-defect migration in Ti-N and Al-N systems. This is motivated by the fact that a number of structural transformations in solid solutions, including spinodal decomposition of B1-Ti_1−x_Al_x_N, is believed to be kinetically controlled by diffusion of lattice vacancies [6,113].

**Figure 7:**
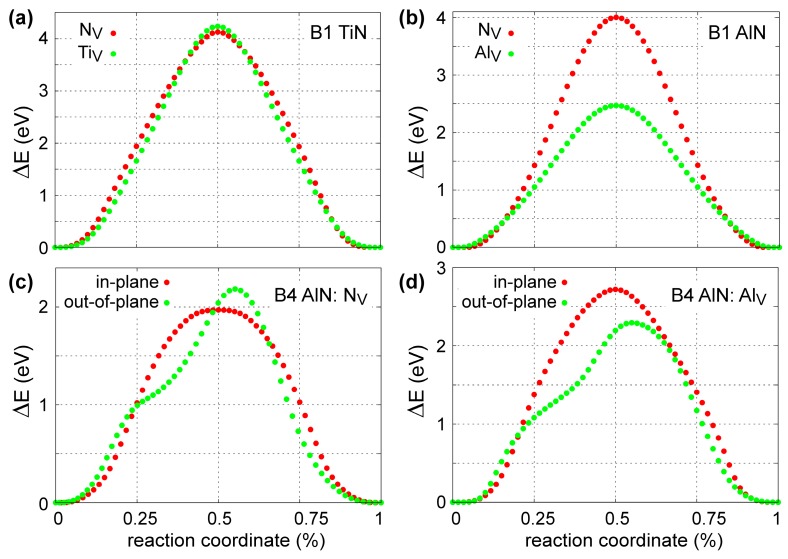
Minimum energy pathways for migration of N (N_V_) and metal (Ti_V_, Al_V_) vacancies among nearest-neighbor sites in the anion (for N_V_) and cation (for Ti_V_ and Al_V_) sublattices in (**a**) B1-TiN, (**b**) B1-AlN, and (**c**,**d**) B4-AlN. For B4-AlN structures, we studied vacancy migration both within, as well as across, the (0001) plane.

Figure 7 presents the results of the 0 K MEAM-NEB calculations for metal (Ti_V_ and Al_V_) and N (N_V_) vacancy migration in B1-TiN (Figure 7a), B1-AlN (Figure 7b), and B4-AlN (Figure 7c,d). We found that the migration energies for Ti_V_ and N_V_ in B1-TiN along <110> directions are 4.13 and 4.24 eV, respectively, with saddle-point transition states located halfway between initial and final atomic positions (Figure 7a). The MEAM predictions are in good agreement with DFT-NEB results, which yield energy barriers of ≈3.8 eV for N_V_ [114] and 4.26 eV for Ti_V_ [97]. Experimental values for vacancy migration energies in B1-TiN are scarce in the literature. Kodambaka et al. [115] determined a global (i.e., both Ti_V_ and N_V_) value of 4.5 ± 0.2 eV for vacancy diffusion energies in bulk B1-TiN. Hultman et al. [116] estimated activation energies for metal interdiffusion at TiN/ZrN superlattice interfaces that range between 2.6 and 4.5 eV, while Wood and Paasche [117] and Anglezio-Abautret et al. [118] reported that activation barriers for N diffusion in B1-TiN lie in the range 1.8 to 5.5 eV.

Besides Ti_V_ and N_V_, we also studied formation and diffusion of Al interstitials (Al_I_) in B1-TiN. Using the energy of an Al atom in fcc-Al as a chemical potential, we calculated that the energy required for forming an Al_I_ at tetrahedral sites is 4.92 eV, which is in reasonable agreement with the DFT value of 3.81 by Mei et al. [96]. Then, the corresponding Al_I_ migration energy across tetrahedral B1-TiN sites was found to be equal to 2.42 eV, which matches perfectly the DFT value from Ref. [96].

Our calculations for N_V_ diffusion within and across the B4-AlN (0001) plane yield activation energies of 1.97 and 2.19 eV, respectively (see Figure 7c). These values are within the experimental uncertainty range of O and N interdiffusion activation energies at Al_2_O_3_/AlN interfaces (2.49 ± 0.42 eV) [119]. For Al_V_, we found that migration across the (0001) B4-AlN lattice planes requires an activation energy of 2.29 eV, which is lower than the value of 2.72 eV for in-plane diffusion (Figure 7d). Moreover, our potential predicts that Al_V_ transport in B1-AlN occurs with an activation energy of 2.47 eV, which is similar to that required in the B4-AlN polymorph. Concurrently, N_V_ migration in B1-AlN requires significantly higher activation energy (4.00 eV) than that in the B4 structure (Figure 7b). We also note that no experimental and/or theoretical data on diffusion of point defects in B1- and B4-AlN are available in the literature to compare with our MEAM results. 

Ab initio data for point-defect diffusion in Ti-Al-N solid solutions are not available in the literature. Nevertheless, experimental studies of structural evolution in annealed B1-Ti_1−x_Al_x_N samples by Mayrhofer et al. [6] and Norrby et al. [120] attributed activation energies of ≈3.3 eV (in Ti_0.36_Al_0.64_N) and ≈3.6 eV (in Ti_0.55_Al_0.45_N) to spinodal decomposition and B1-to-B4 transformations within AlN-rich domains, which in turn, is primarily attributed to diffusion of metal and nitrogen vacancies. The experimental estimates of atomic migration energies during spinodal decomposition in Ti-Al-N (3.3–3.6 eV) are consistent with the range of values that we obtain for cation and anion diffusion in binary Ti-N and Al-N. 

Although determination of point-defect formation and migration energies in Ti-Al-N solid solutions is strongly dependent on the local chemical environment [121] (and therefore lies outside the scope of this work), we used CMD simulations to ensure stability of B1-Ti_1−x_Al_x_N alloys containing defects, i.e., monovacancies, divacancies, interstitials, and interstitial pairs at different temperatures. We found that point defect migration and point-defect/point-defect interactions do not cause unphysical structural transformations within the alloys during the investigated time scales, which are of the order of one nanosecond. This, together with the results presented above, lends confidence that our model potential is suitable to investigate phase transformation phenomena in Ti-Al-N solid solutions.

### 4.4. Equilibrium Volumes and Elastic Properties of B1-TiN_y_, B2-TiN, and bct-Ti_2_N

The proposed set of MEAM parameters (see Supplemental Material) was carefully fitted to the properties of stoichiometric B1-TiN and ε-Ti_2_N. To verify transferability of our model to a variety of Ti-N lattice configurations and bonding geometries, which may be encountered during simulations of dynamic processes in Ti-Al-N alloys, we present here the elastic and structural properties calculated for nitrogen-deficient B1-TiN_y_ (0.69 ≤ y < 1) as well as high-pressure B2-TiN and bct-Ti_2_N phases.

**Figure 8:**
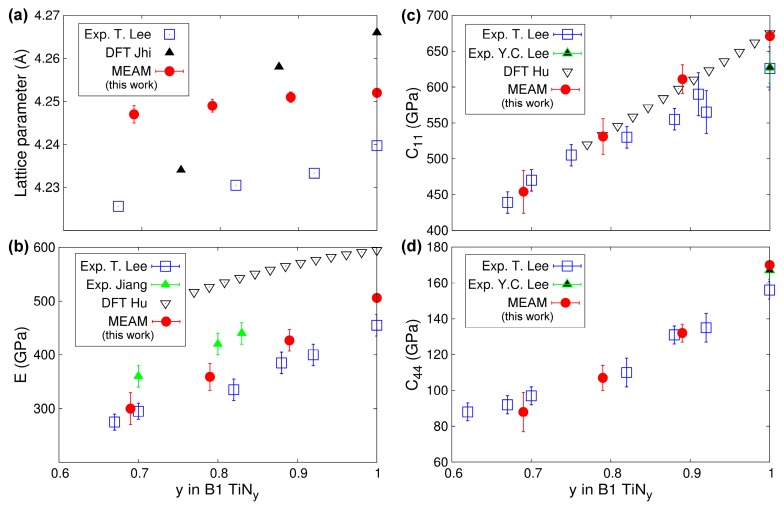
MEAM TiN_y_ (0.69 ≤ y ≤ 1) lattice parameters and elastic constants as function of N content y calculated at 0 K in comparison with experimental (T. Lee = Ref. [84], Y.C. Lee = Ref. [85], Jiang = Ref. [128]), and ab initio (DFT Jhi = Ref. [126], DFT Hu = Ref. [127]) results.

Ti-N compounds maintain the cubic B1 structure over a wide compositional range [122]. In B1-TiN_y_, understoichiometry (y < 1) is primarily accommodated by anion vacancies [123]. In addition, control of the N content during synthesis can be used to tune the TiN_y_ optical [124], electrical [106], and mechanical [84,125] properties. In Figure 8, we plot the lattice parameter a_TiNy_ (Figure 8a), Young’s modulus *E* (Figure 8b), and elastic constants *C_11_* and *C_44_* (Figure 8c,d, respectively) as predicted using 0 K calculations with our MEAM potential along with experimental and DFT data for comparison. We observe (Figure 8a) that a_TiNy_ increase monotonically with increasing the N content y, in excellent agreement (maximum discrepancy ≈0.5%) with experimental measurements [84]. Remarkably, our MEAM potential reproduces the experimental a_TiNy_ versus y trend better than DFT [126]. Other results (not included in Figure 8a) show that, depending on the choice of the electronic exchange-correlation approximation, DFT overestimates or underestimates the lattice parameter of stoichiometric TiN by up to 1.3% [36]. DFT calculations [126,127] and experiments (acoustic wave velocities and nanoindentation tests [84,85,128]) have demonstrated that *E*, *C_11_*, and *C_44_*, in B1-TiN_y_ increase monotonically as function of y, as shown in Figure 8b–d. Our MEAM results reproduce the above-described trend and specific values of the elastic constants well.

Additional MEAM calculations were carried out to assess basic properties of B2-TiN and bct-Ti_2_N phases for comparison with experimental and ab initio results. For bct-Ti_2_N, our model yields lattice constants a = 4.152 Å and c = 8.930 Å, in excellent agreement with experimentally-determined ranges of a = 4.140–4.198 Å and c = 8.591–8.805 Å [73], and DFT values of a = 4.151 Å and c = 8.880 Å [129]. The elastic properties determined by MEAM for the bct-Ti_2_N phase were consistent with DFT values (given in the parenthesis) as explained in the following: *B* = 177 (179) GPa, *C_11_* = 456 (372) GPa, *C_33_* = 207 (287) GPa, *C_12_* = 205 (126) GPa, *C_13_* = 120 (87) GPa, *C_44_* = 45 (70) GPa, and *C_66_* = 125 (109) GPa. For the cubic B2 TiN-phase, MEAM yields a lattice parameter of 2.717 Å and a bulk modulus of 256 GPa versus a = 2.638 Å and *B* = 249 GPa calculated via DFT (present work) employing hard (i.e., optimized to model high-pressure properties) PBE exchange-correlation functionals.

### 4.5. Phase Stability and Transitions

#### 4.5.1. Ti-N

The cohesive energies of B1-TiN and ε-Ti_2_N phases, which are the ground-state configurations for the TiN and Ti_2_N systems, were reproduced via the ASA parametrization (see Section 3 and Table 1). Post-parametrization calculations were carried out to verify that our model predicted correct trends in the energetics for high-pressure metastable B2-TiN and bct-Ti_2_N polymorph structures. The MEAM potential predicts that the B1-TiN structure was ≈1.6 eV/atom more stable than the B2-TiN phase, in fair agreement with present DFT calculations which yield an energy difference of ≈0.9 eV/atom between the two phases. Moreover, we find that ε-Ti_2_N is 98 meV/atom more stable than the bct-Ti_2_N polymorph, which is qualitatively consistent with DFT results that have shown an energy difference of 16 meV/atom in favor of the ε-Ti_2_N structure [129].

#### 4.5.2. Al-N

At atmospheric pressure and for *T* = 300 K, B4 is the thermodynamically stable AlN structure. However, subject to compression, polycrystalline B4-AlN samples transform into the metastable B1-AlN polymorph [67,74,130]. Experiments indicate that the B4-to-B1 transition pressure decreases from ≈14 GPa (at 300 K) to ≈11–12 GPa at temperatures between 1000 and 2000 K [67,74,130]. For comparison, DFT-based studies report a transition pressure that decreases monotonically with increasing *T* from ≈13–17 GPa at 0 K to ≈6 GPa at 3000 K [60,77,131]. Other first-principles investigations based on the quasi-harmonic approximation [132] demonstrate that the B4- to B1-AlN transition pressures predicted via DFT strongly depend on the approximation used for the electronic exchange-correlation energy: the B4/B1 phase boundary calculated ab initio with two different exchange-correlation functionals decreases, for temperatures increasing from 0 to ≈2200 K, from 7 to 5 GPa and from 12 to 10 GPa (figure 2 in Ref. [132]). Moreover, the AlN phase diagram obtained via AIMD simulations indicates that the transition pressure changes from 13 GPa (at 0 K) to 0 GPa (at 3200 K) (see figure 1 in Ref. [50]). The free energies of B1- and B4-AlN calculated as a function of temperature and pressure via CMD (see Section 2.2 for details) show that, at 300 K, the B1-AlN polymorph structure becomes the most thermodynamically stable at a pressure of ≈5 GPa, which is quite below the experimentally-determined value (≈14 GPa). CMD results yield a B4/B1 phase boundary that remains near ≈4 GPa up to the AlN melting point estimated by MEAM (*T_m_* ≈ 3200 K). This is consistent with the result that MEAM underestimates the energy difference *E_c,B4-AlN_* – *E_c,B1-AlN_* (see Table 1).

**Figure 9:**
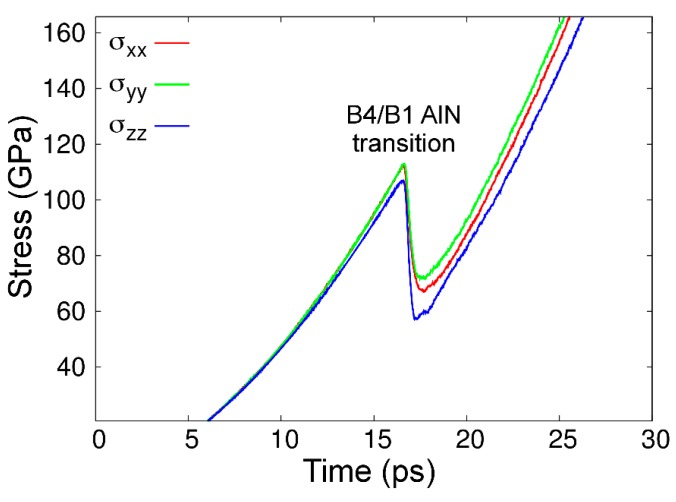
Stress as a function of time/pressure in the B4-AlN simulation box during the CMD runs. The abrupt drop in stress at ≈17 s (≈110 GPa) corresponding to the formation of small B1-AlN grains, which is the first step for the B4- to B1-AlN phase transformation.

Despite the fact that the AlN phase diagram based upon CMD free-energy calculations is only in reasonable agreement with experimental and ab initio findings, our model reproduces the correct dynamics of the B4-to-B1 AlN transition. This is seen in Figure 9, which shows the evolution of stress in a B4-AlN crystal along the three orthogonal directions upon applying external pressure that increases at an average rate of ≈7 GPa·ps^–1^, at 300 K (see more details in Section 2.2). After a monotonic increase up to a value of 110 ± 10 GPa, the stress accumulated in B4-AlN is partially relieved. As we discuss in detail below, this stress relief is due to the formation of small B1-AlN grains. 

The pressure value at which the B4- to B1-AlN phase transition was initiated in our CMD simulations is in excellent agreement with the results of AIMD modeling (100–120 GPa, see figure 1 in Ref. [133]). The B4-to-B1 AlN transition pressure values obtained during present CMD and previous AIMD [133] simulations at 300 K are considerably above the interval of pressures (5–14 GPa) predicted in our AlN phase diagram, as well as in AIMD and experimental AlN phase diagrams [50,74]. This is an expected discrepancy; while CMD simulations are carried out for defect-free crystals during extremely short times (≈10^–10^ s), compression experiments are conducted at close-to-equilibrium conditions (≥10^2^ s) and on polycrystalline B4-AlN samples. Hence, pressures much larger than those predicted by thermodynamics (phase diagrams) are necessary to quickly activate the B4-to-B1 AlN transition during the timescales accessible via molecular dynamics simulations. The fact that our CMD results (Figure 9) match AIMD predictions [133] suggests that the kinetic free-energy barrier that separates the B4- and B1-AlN phases is accurately reproduced by MEAM.

**Figure 10:**
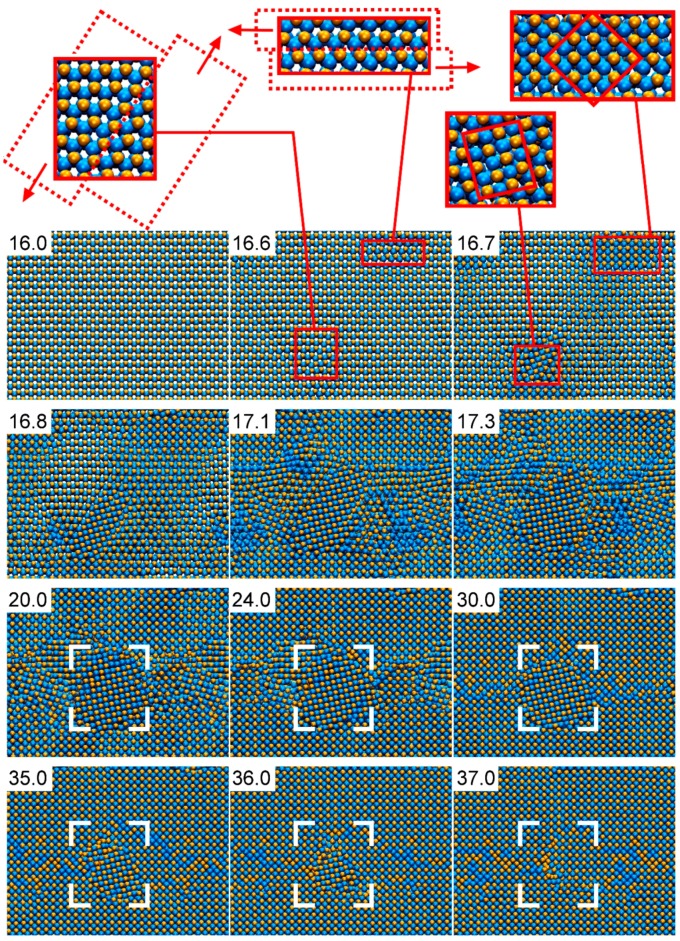
CMD simulation snapshots of the B4- to B1-AlN phase transformation induced by pressure. B4-AlN is seen from the [0001] direction. See text for details and explanation of the features observed in the figure.

Figure 10 presents the detailed structural evolution observed in AlN under compression during CMD simulations (full movie available in the Supplementray Materials, Video S1). The defect-free B4-AlN structure is retained up to a pressure of ≈100 GPa (simulation time of 16 ps). The occurrence of local lattice slip at a pressure of ≈110 GPa (shown in the magnified B4-AlN regions at 16.6 ps in Figure 10) leads to the formation of small B1-AlN grains, as seen in the insets of the snapshot for 16.7 ps. This causes a pressure drop from ≈110 to ≈70 GPa. From that point, B1-AlN grains continue growing at the expense of the B4-AlN crystal. At approximately 20 ps (≈85 GPa), a single phase polycrystalline B1-AlN structure is formed. Thereupon, smaller B1-AlN grains coalesce with the larger ones, see e.g., the areas enclosed in the white brackets in the simulation snapshots corresponding to times between 24 and 36 ps (≈140–330 GPa). At a simulation time of ≈37 ps (≈350 GPa), the original defect-free B4-AlN has transformed into defective B1-AlN crystal. The B1-AlN structure is retained up to pressures of ≈1500 GPa (not shown in Figure 10), which is the largest pressure used in our simulations. This result is consistent with X-ray diffraction observations, which excluded the occurrence (at equilibrium conditions) of a second solid-to-solid AlN phase transition at pressures up to 132 GPa [79].

#### 4.5.3. Ti-Al-N

B1-Ti_1−x_Al_x_N solid solutions with high (x > 0.5) AlN contents are known to decompose via the spinodal route, at temperatures near 1200 K [7,8], leading to formation of coherent Ti- and Al-rich B1-Ti_1−x_Al_x_N domains. This hinders dislocation motion across strained domain interfaces and results in the age-hardening effect, which is of extreme importance for metal cutting at elevated temperatures [134,135]. A further increase in temperature causes B1-to-B4 structural transformation within Al-rich domains, which is detrimental for hardness and coating performance [7,8,134,135]. To date, the atomistic mechanisms that control spinodal decomposition and subsequent formation of B4-AlN in Ti-Al-N coatings are unclear. These processes can be elucidated by means of large-scale CMD simulations using our MEAM potential. The suitability of the potential for such simulations is explored in this section by discussing the B1-Ti_1−x_Al_x_N phase diagram predicted using MEAM, in comparison to first-principle and experimental results. 

**Figure 11:**
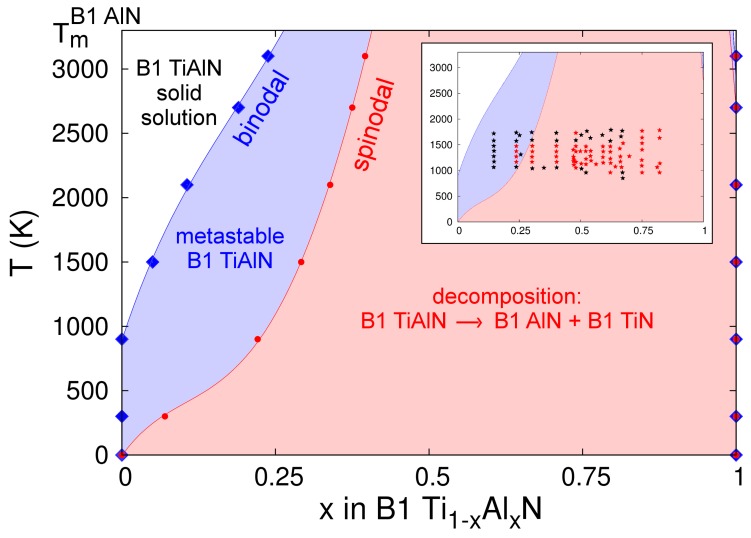
Spinodal and binodal curves for B1-Ti_1−x_Al_x_N solid solutions calculated using CMD free energies of mixing with respect to B1-TiN and B1-AlN. The inset compares our results to experimental data (black and red stars mark B1-Ti_1−x_Al_x_N and spinodally-decomposed solid solutions, respectively) collected in Ref. [138].

The B1-Ti_1−x_Al_x_N binodal and spinodal curves calculated on the basis of CMD free energies of mixing are presented in Figure 11. It is important to note that our phase diagram ends at the AlN melting point (*T_m_* ≈ 3200 K); the temperature beyond which the free-energy of pure B1-AlN (necessary to compute the alloy free energy of mixing) is not defined. This, however, does not imply that B1-AlN-rich regions may not form within B1-Ti_1−x_Al_x_N solid solutions during CMD simulations at *T* > 3200 K. Our estimated binodal and spinodal regions, Figure 11, are in very good agreement with recent thermodynamic assessments (see figure 2 in Ref. [136]), as well as ab initio calculations accounting for vibrational effects on alloy free energies (see figure 3 in Ref. [93]). Our CMD results also match reasonably well with the Ti-Al-N phase diagram determined via AIMD simulations (see figure 2 in Ref. [137]).

Previous DFT calculations, in which temperature effects on alloy mixing free energies were neglected or added as mean-field theory corrections to mixing enthalpies determined at 0 K [3,91,92], estimated a consolute temperature *T_c_* in the range ≈7500–9000 K. Subsequent first-principles investigations by Wang et al. [93] showed that accounting for vibrational entropies *S_vib_* in Ti-Al-N free energies of mixing via the Debye–Grüneisen approximation significantly lowered the calculated *T_c_* (≈3800 K). More recently, AIMD results by Shulumba et al. demonstrated that the explicit inclusion of anharmonic effects in *S_vib_* further reduces the predicted *T_c_* to 2900 K [137]. Consistent with the observations of Refs. [93,137], our CMD-based evaluations also show that the implicit inclusion of *S_vib_* yields narrower miscibility gaps at temperatures above ≈1500 K, as compared to those close to room temperature. 

At elevated temperatures, the CMD phase diagram presented in Figure 11 exhibits discrepancies with previous ab initio [93,137] and Calculation of Phase Diagrams (CALPHAD) [136] results. For example, at a temperature of 2500 K, the spinodal region predicted by the MEAM potential spans in the AlN content x range 0.35 to 1, while Refs. [93,136,137], indicate spinodal regions for 0.4 < x < 0.9, 0.4 < x < 0.9, and 0.6 < x < 0.9, respectively. Nonetheless, consistent with several first-principles results [3,91,92,93,136,137], our Ti-Al-N phase diagram is skewed toward the AlN end, indicating that TiN-rich alloys are more stable than their AlN-rich counterparts. In stark contrast with the predictions of our model and Refs. [3,91,92,93,136,137], another recent thermodynamic evaluation [138] reports a B1-Ti_1-x_Al_x_N phase diagram which is skewed toward the TiN end and with the lowest *T_c_* (≈2100–2200 K) calculated so far (see figure 1 in Ref. [138]). 

In their paper [138], Zhou et al. provide a detailed overview of experimental results collected during the past two decades for annealed B1-Ti_1-x_Al_x_N solid solutions. These are included for comparison with our results in the inset of Figure 11, where black stars denote stable or metastable cubic alloy compositions, and red stars mark spinodally-decomposed Ti-Al-N samples. A first apparent inconsistency between theoretical and experimental assessments of miscibility gaps can be seen for *T* ≤ 1000 K; the experimental observations (black stars) indicate stability (or metastability) of B1-Ti_1−x_Al_x_N alloys for AlN concentrations well within the spinodal compositional range (0.25 < x < 1) predicted by the CMD phase diagram. Refractory metal-nitride compounds exhibit relatively low atomic mobilities [139], which may shift the onset of structural transformation to temperatures larger than those predicted in theoretical models. In order to explore possible effects of low point-defect mobilities on the outcome of annealing experiments conducted on Ti-Al-N, we consider the case of the parent compound TiN, for which nitrogen diffusivities *D*(*T*) are available in the literature [36] (in TiN, Ti migration is even slower than N migration [97]). We consider the model system B1-TiN_1–y_ with nitrogen vacancy concentrations at the dilute limit (y ≈ 10^–5^) and diffusivities *D*(1100 K) ≈ 10^–20±2^ cm^2^·s^–1^ and *D*(800 K) ≈ 10^–33±2^ cm^2^·s^–1^, as shown in figure 11 of Ref. [36]. Annealing B1-TiN_1−y_ at a temperature *T* = 1100 K during one day (t ≈ 10^5^ s) would induce nitrogen transport over distances d ≈ [6·t·*D*(1000 K)]½ of a few nm. A modestly lower annealing temperature (800 K) would require considerably longer times (years) to observe diffusion over comparable length scales. Given the similarity in chemical bonds and crystal structures, it is reasonable to assume that nitrogen and metal atoms in Ti-Al-N migrate at rates comparable to those discussed above for TiN, thus providing a possible explanation for the retained stability of B1-Ti_1−x_Al_x_N solid solutions at *T* ≤ 1000 K (inset in Figure 11). 

The experimental results are in consistent with theory for temperatures between 1000 K and ≈1500–1800 K: (i) no decomposition was observed for x < ≈0.2 (black stars); (ii) spinodal regions (red stars) are within the range ≈0.25 < x < ≈0.8. Nonetheless, for *T* > 1700 K, the experimental observations [138] indicate that Ti-Al-N solid solutions with AlN contents in the range ≈0.25 < x < ≈0.6 maintain the cubic structure. At such high temperatures, the scenario of spinodal decomposition being kinetically limited is not valid. This may indicate that the miscibility gap closes at temperatures as low as ≈1700 K, which is well below the consolute temperature predicted in all theoretical TiAlN phase diagrams (including the one in the present study) reported in the literature.

## 5. Summary and Outlook

In this work, we presented a MEAM interatomic potential for the Ti_1−x_Al_x_N (0 ≤ x ≤ 1) alloy system. This was achieved by developing an adaptive simulated annealing (ASA) algorithm for scanning the MEAM multi-dimensional parameter space and identifying a set of parameters that optimize the potential’s predictions relative to 0 K experimentally- and ab-initio-determined equilibrium volumes, cohesive energies, elastic constants, defect formation energies, and mixing enthalpies of elemental, binary, and ternary phases and configurations within the Ti-Al-N system. We then validated the transferability of the parameters selected via the ASA procedure by establishing the ability of the potential to reproduce known finite-temperature kinetic and thermodynamic properties, not explicitly included in the ASA optimization, for B1-Ti_1−x_Al_x_N (0 ≤ x ≤ 1), B4-AlN, and various Ti-N phases. We found that, overall, the potential reproduces the following well: (i) temperature-dependence of equilibrium volumes in B1-Ti_1−x_Al_x_N and B4-AlN; (ii) phonon dispersion curves of B1-TiN, B1-AlN, and B4-AlN; (iii) point-defect migration energies in B1-TiN, B1-AlN, and B4-AlN; (iv) lattice and elastic constants of sub-stoichiometric B1-TiN_y_ (0.7 ≤ y ≤ 1), B2-TiN, and bct-Ti_2_N; (v) free energies of B1-AlN and B4-AlN, and dynamics of B4- to B1-AlN pressure-induced phase transformation; and (vi) B1-Ti_1−x_Al_x_N alloy mixing free energies and binodal/spinodal phase boundaries with respect to B1-TiN and B1-AlN.

The outcome of the present study is an interatomic potential that can enable large-scale classical molecular dynamics simulations in one of the most important engineering materials systems, i.e., Ti-Al-N. These simulations may include thermal stability of B1-Ti_1−x_Al_x_N solid solutions, multilayers, and self-organized nanostructures and can provide critical insights onto the atomistic pathways that drive segregation via the spinodal route and phase transformation via nucleation and growth. Our MEAM potential can also be used to simulate elastic and plastic responses of B1-Ti_1−x_Al_x_N alloys, which together with thermal stability simulations can guide the design of protective coatings with superior mechanical and oxidation performance. Other areas where our MEAM set of parameters can be used include diffusion of Al in B1-Ti_1−x_Al_x_N used as barrier layer and contact material in microelectronic devices [140,141,142]. Moreover, the present set of MEAM parameters can be used as starting point for developing potentials that can simulate vapor-based growth of Ti-Al-N films. Beyond Ti-Al-N, the methodologies presented in this work can serve as the foundation for developing potentials for quaternary systems, e.g., Ti-Al-Si-N, Ti-Al-Ta-N, and Ti-Al-Nb-N, where complex metal-sublattice configurations are used to enhance surface wear and oxidation resistance, and inhibit wurtzite-phase formation subsequent to spinodal decomposition, which allows extending the age hardening effect to temperatures above 1200 K [143,144,145,146,147,148,149,150].

## Figures and Tables

**Table 1:** MEAM-predicted cohesive energies (*E_c_*), lattice constants (a, c), and elastic constants (*B*, *C*_ij_) for B1-TiN, ε-Ti_2_N, B1-AlN, B3-AlN, and B4-AlN. Experimental and DFT values are listed in brackets and parenthesis, respectively.

	B1-TiN	ε-Ti_2_N	B1-AlN	B3-AlN	B4-AlN	
***E_c_***	6.613	6.180	5.690	5.728	5.758	
**(eV/at.)**	[6.69 ± 0.07^c1^] (6.8^d1^, 8.708^c1^)		(5.597^e1^)	(6.621^e^, 5.681^e^, 5.44^f^)	[5.76^g^, 5.76^o^] (5.701^e^, 5.779^e1^, 6.643^e^, 5.055^r^, 5.545^r^)	
**Energy above hull** **(meV/at.)**			68	30	0	
	-	-	(147^q^, 204^a^, 172^c^, 182^e1^)	(43^q^, 23^a^, 21^b^, 21^e^, 22^e^, 41^f^)		
***a***	4.252	4.939	4.090	4.366	3.112	
**(Å)**	[4.240^z^] (4.188–4.254^s^)	[4.938–4.946^l^] (4.955*, 4.960^j^)	[4.046^u^, 4.064^w^] (4.014–4.070^v^, 4.06^a^, 4.069^c^, 4.071*)	[4.37^d^, 4.38^p^] (4,349^y^, 4.39^a^, 4.320^x^, 4.401^b^, 4.310^e^, 4.394^e^, 4.374^f^)	[3.111^d^, 3.111^b1^, 3.110–3.113^f^] (3.12^a^, 3.06^x^, 3.100^f^, 3.113^e^, 3.057^e^, 3.129^k^, 3.101^r^, 3.117^r^)	
**c/a**		0.616			1.600	
	-	[0.613–0.614^l^] (0.612^j^, 0.613*)	-	-	[1.601^d^, 1.600^b1^, 1.602^h^, 1.600–1.602^f^] (1.596^q^, 1.603^a^, 1.60^x^, 1.619^e^, 1.617^e^, 1.609^f^, 1.603^k^, 1.598^r^, 1.604^r^)	
***B***	298	208	277	237	236	
**(GPa)**	[298–324^a1^] (277^n^, 303^t^, 290–350^s^)	(204^j^, 214*)	[221 ± 5^m^, 295 ± 17^u^, 319 ± 8^w^] (253–277^v^, 270^q^, 207^n^, 265^t^, 255^c^, 261*)	[202^p^] (213^y^, 216^q^, 195^b^, 206^e^, 209^x^, 191^e^, 218^f^, 228^r^)	[208 ± 6^h^, 211^b1^, 185 ± 5^m^, 185–212^f^, 185–237^o^, 303 ± 4^w^] (205^q^, 202^x^, 209^e^, 192^e^, 194^k^, 228–243^r^)	
***C*_11_**	613	309	480	289	432	
**(GPa)**	[626^z^, 605–649^a1^] (590^n^, 610^t^, 640–710^s^)	(429^j^, 434*)	(340^n^, 425^t^, 428^c^, 432*)	[328^p^] (309^y^, 284^b^, 298^x^, 348^r^)	[395^p^, 411^b1^, 410 ± 10^i^, 345–411^o^] (458^x^, 376^k^, 389–464^r^)	
***C*_12_**	140	153	175	213	203	
**(GPa)**	[145–165^a1^] (120^n^, 150^t^, 115–125^s^)	(105^j^, 127*)	(140^n^, 185^t^, 168^c^, 175*)	[139^p^] (164^y^, 150^b^, 164^x^, 168^r^)	[125^p^, 149^b1^, 148 ± 10^i^, 125–149^o^] (154^x^, 129^k^, 149–158^r^)	
***C*_44_**	165	130	271	100	70	
**(GPa)**	[156^z^, 162–171^a1^] (160^n^, 165^t^, 159–169^s^)	(151^j^,169*)	(260^n^, 298^t^, 307^c^, 296*)	[133^p^] (78^y^, 179^b^, 187^x^,135^r^)	[118^p^, 125^b1^, 125 ± 5^i^, 118–125^o^] (85^x^, 113^k^)	
***C*_13_**		163			153	
**(GPa)**	-	(194^j^, 205*)	-	-	[120^p^, 99^b1^, 99 ± 4^i^, 95–120^o^] (84^x^, 98^k^, 116–138^r^)	
***C*_33_**		296			337	
**(GPa)**	-	(300^j^, 337*)	-	-	[345^p^, 389^b1^, 388 ± 10^i^, 394–402^o^] (388^x^, 353^k^, 408–409^r^)	
***C*_66_**		121			115	
**(GPa)**	-	(138^j^, 136*)	-	-	[135^p^, 131^b1^, 131 ± 10^i^, 130–131^o^] (152^x^, 124^k^, 115–157^r^)	

* present work, DFT with generalized gradient approximation (see Supplementary Materials), a = [60], b = [61], c = [62], d = [63], e = [64], f = [65], g = [66], h = [67], i = [68], j = [69], k = [70], l = [73], m = [74], n = [71], o = [75] and references therein, p = [76], q = [77], r = [78], s = [36], t = [72], u = [79], v = [80], w = [81], x = [82], y = [83], z = [84], a1 = [85], b1 = [86], c1 = [88], d1 = [89], e1 = [87].

## Supplementary Materials

The following are available online at http://www.mdpi.com/1996-1944/12/2/215/s1, Text S1: Supplemental material “Semi-empirical force-field model for Ti1-xAlxN (0 ≤ x ≤ 1) system” which includes details on the MEAM formalism, the ASA parametrization procedure, and the parametrization, validation results for the N, Ti, Al, and Al-Ti phases, and a list of the MEAM parameters (elaborated in the respective sections therein and Tables S1–S3 and Figures S1–S7), Video S1: Visualization of B4- to B1-AlN phase transformation upon pressure change; Script S1: Fortran scripts for: (i) creating a defect-free B1 supercell with axes oriented along [100], [010], and [001] with desired number of replicas in the three directions; and (ii) randomization of the atomic positions on one sublattice.
