# Supplementary material for: Semi-Empirical Force-Field Model for the Ti1−xAlxN (0 ≤ x ≤ 1) System
Source: Materials (Basel). 2019 Jan 10;12(2):215. doi: 10.3390/ma12020215 (PMC6356630; doi:10.3390/ma12020215)
Supplement: Supplementary file 1 [file materials-12-00215-s001.zip › materials-404605-SI.pdf]

materials

MDPI

Supplemental Material

S1. MEAM Formalism

The second-nearest neighbor MEAM potential formalism employed in the present work allows both positive and negative background density values. We employ a G-function with form $G(\Gamma) = \begin{cases} +\sqrt{|1+\Gamma|}, & \Gamma \geq -1 \\ -\sqrt{|1+\Gamma|}, & \Gamma < -1 \end{cases}$, an equation of state $E^u(R) = -E_c\left(1 + a^* + \frac{d_{+|-}a^{*3}}{r/r_e}\right)e^{-a^*}$ with $a^* = a\left(\frac{r}{r_e} - 1\right)$ and an ialloy LAMMPS parameter = 0. A detailed description of MEAM/LAMMPS parameters and relevant references for the MEAM formalism can be found in Ref. [1].

The global potential cutoff distance is set to r_c = 5.10 Å to take into account atomic interactions up to (at least) the second neighboring shell for every investigated phase, with a smoothing cutoff distance of Δr = 0.36 Å. For all MEAM-reference elemental and binary structures (N_2 dimer, bcc-Ti, fcc-Al, $L1_2$-Al_3Ti, B1-TiN, and B1-AlN), the ranges of the MEAM parameters E_c, r_e, α, d_+, and d_- in the ASA optimization procedure (see Section 2.1 of the paper and Section S2 in the present document) are constrained close to values that best fit energy vs. volume curves obtained density functional theory (DFT) calculations with different electronic exchange-correlation functionals (PBE, LDA, BLYP, B1LYP, B3LYP, and BHandHLYP), see Figures S1 and S2. The determination of E_c by DFT entails calculating the energies of isolated N, Al, and Ti atoms to be subtracted from reference-structures self-consistent energies. DFT is carried out with the VASP code [2] implemented with the projector augmented wave (PAW) [3] method. For all structures, cohesive energies are converged to an accuracy of 10^{-5} eV/atom with respect to k-point grid thicknesses and planewave energy cutoffs.

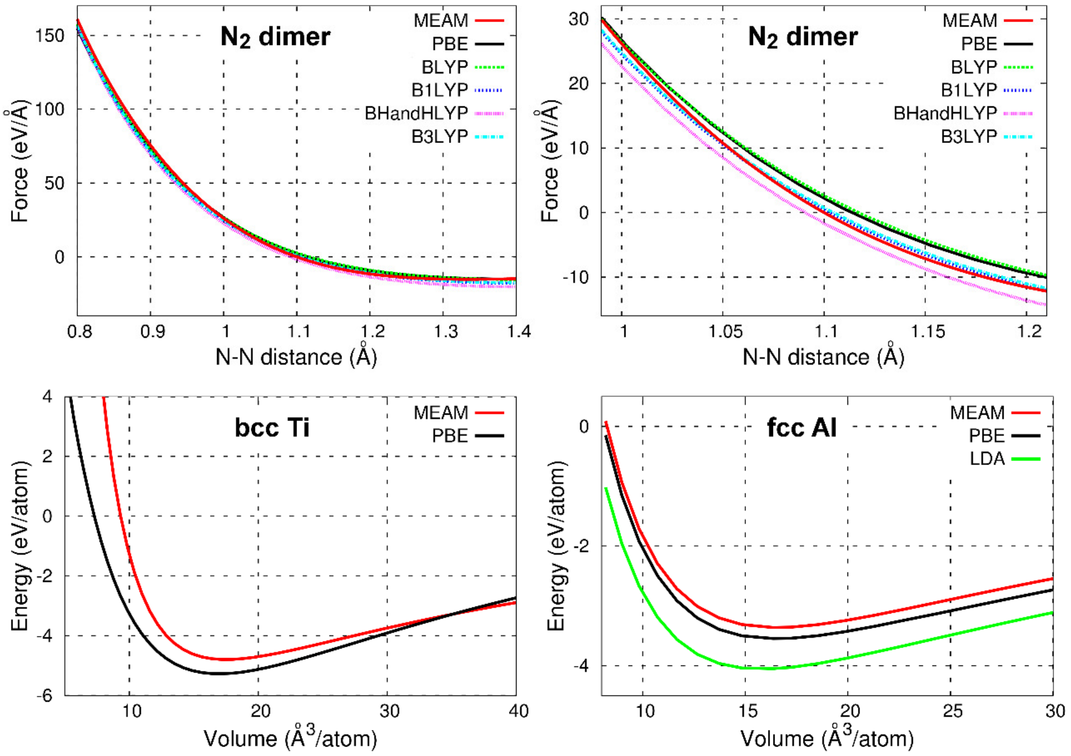

Figure S1. MEAM vs. ab initio energy vs. volume curves calculated for chosen elemental reference structures. Top panels: Interatomic forces in N_2 dimer with magnification near N-N equilibrium distance. Bottom panels: bcc Ti and fcc Al. *Ab initio* N_2 interatomic forces are taken from Ref. [4]. MEAM curves are fitted to the DFT curves by adjusting the values of the MEAM parameters E_c, r_e, α, d_+, and d_-.

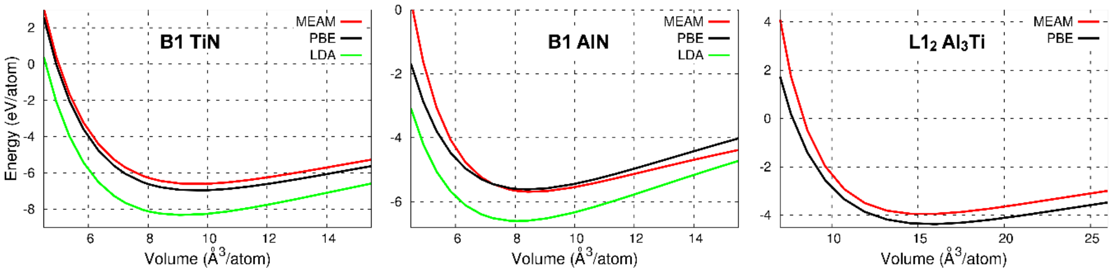

Figure S2. MEAM vs. ab initio energy vs. volume curves calculated for chosen binary reference structures: B1 TiN, B1 AlN, and $L1_2$ Al_3Ti. MEAM curves are fitted to the DFT curves by adjusting the values of the MEAM parameters E_c, r_e, α, d_+, and d_-.

S2. Adaptive Simulated Annealing (ASA) Parametrization Procedure

The flow chart of the Metropolis algorithm combined with adaptive simulated annealing (ASA) is illustrated in Figure S3. This algorithm enables us to efficiently sample the multidimensional MEAM parameter space and identify candidate sets of parameters that fit experimentally- and DFT-determined physical properties of reference phases and structures as explain in Section 2.1 of the paper. The set of MEAM parameters that best optimizes the potential predictions, and is successfully validated via CMD simulations of material thermodynamic properties (see Section 2.2 of the paper), is listed in Table S1 at the end of this document. We strongly encourage interested users *not to transcribe* the parameters to from Table S1 but rather directly download source files in LAMMPS format from online repositories [5,6].

Table S1. The complete set of the MEAM parameters for the ternary Al-Ti-N system. A global potential cutoff of r_c=5.10 Å and a smoothing cutoff distance of Δr=0.36 Å are applied. In the table header row, elements are given in order of type-number for LAMMPS input files.

	N	Al	Ti	N-Al	N-Ti	Al-Ti	N-Al-Ti
Ref. Structure	dimer	fcc	bcc	B1	B1	$L1_2$	–
E_c (eV)	4.880	3.357	4.798	5.721	6.614	3.956	–
r_e (Å)	1.100	2.86166	2.832	2.042	2.126	2.788	–
α	4.680	4.692	4.799	4.757	4.930	5.021	–
d_+	0.00	0.10	0.09	0.17	0.03	0.03	–
d_-	-0.02	0.03	0.10	0.09	0.03	0.08	–
ρ_0	23.80	1.00	1.02	–	–	–	–
A	1.51	1.07	0.71	–	–	–	–
$b^{(0)}$	2.55	3.47	2.36	–	–	–	–
$b^{(1)}$	2.28	3.92	0.75	–	–	–	–
$b^{(2)}$	3.68	2.74	0.78	–	–	–	–
$b^{(3)}$	3.92	0.30	0.33	–	–	–	–
$t^{(0)}$	1.00	1.00	1.00	–	–	–	–
$t^{(1)}$	0.39	5.43	3.94	–	–	–	–
$t^{(2)}$	-0.23	9.32	8.09	–	–	–	–
$t^{(3)}$	-0.40	-4.57	-19.47	–	–	–	–
C_{min} [(1,1),1]	1.72	0.92	0.85	–	–	–	–
C_{max} [(1,1),1]	3.49	1.66	2.06	–	–	–	–
C_{min} [(1,1),2]	–	–	–	0.46	0.86	0.60	–
C_{max} [(1,1),2]	–	–	–	4.00	3.14	1.47	–
C_{min} [(1,2),2]	–	–	–	0.31	0.85	0.40	–
C_{max} [(1,2),2]	–	–	–	0.68	2.08	1.88	–
C_{min} [(1,2),1]	–	–	–	0.01	0.25	0.70	–
C_{max} [(1,2),1]	–	–	–	2.62	2.57	1.59	–
C_{min} [(2,2),1]	–	–	–	0.18	0.02	1.84	–

C_{max} [(2,2),1]	–	–	–	3.13	1.63	3.63	–
C_{min} [(1,2),3]	–	–	–	–	–	–	2.16
C_{max} [(1,2),3]	–	–	–	–	–	–	2.84
C_{min} [(1,3),2]	–	–	–	–	–	–	0.39
C_{max} [(1,3),2]	–	–	–	–	–	–	0.95
C_{min} [(2,3),1]	–	–	–	–	–	–	0.04
C_{max} [(2,3),1]	–	–	–	–	–	–	1.69

The N-dimensional parameter space is sampled according to Boltzmann statistics based on an arbitrary cost-function χ. The cost function is chosen *a priori* and is equal to the summation of squared differences between predicted and reference properties. χ is the quantity that is to be minimized in order to determine candidate sets of reliable parameters. ASA optimization is initialized with relatively high dummy-temperature T values. Then, T is progressively reduced with time t as: $T(t) = T(0)\cdot\exp[-a\cdot(t^{b/N})]$, where t is the Metropolis Monte-Carlo timestep, a and b are freely adjustable variables, and N is the number of parameters to be optimized via ASA. The strategy of modifying T allows for (*i*) at high T: escaping from local χ minima, (*ii*) at lower T: selectively exploring regions of the parameter space characterized by low χ.

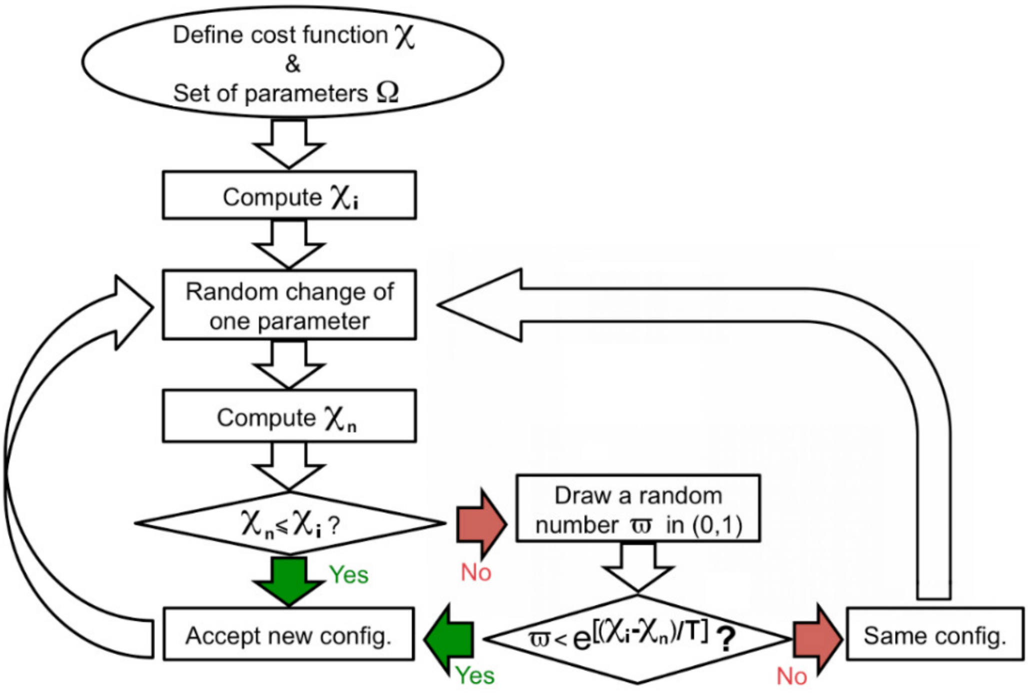

Figure S3. Flowchart of Metropolis algorithm implemented within an adaptive simulated annealing scheme (dummy temperature T).

S3. Parameterization and validation results for N, Ti, Al, and Al-Ti phases

Given that basic MEAM parameters for pure N are predetermined by fitting first principles results for N_2 molecules (Figure S1), the MEAM parameters of elemental N are further optimized via the ASA scheme with respect to *ab initio* properties calculated for N_3 trimers (Table S2).

Table S2. Cohesive energies, and interatomic distances d_{ij}, d_{jk} calculated using the present potential for three stable (or metastable) nitrogen configurations. Reference values used for the parameterization (in parenthesis) were determined via DFT calculations.

	**N_2 Dimer**	**N_3 Linear Trimer**	**N_3 Triangular Trimer**
E_c	4.880	4.063	3.887
(eV/at.)	(4.88)	(4.146)	(3.676)
$<d_{ij}>$	1.10	1.15	1.48
(Å)	(1.10)	(1.20)	(1.40)
d_{ik}/d_{jk}	–	1.980	1.800
		(2.000)	(1.154)

Table S3 lists the predictions of our MEAM potential for the cohesive energies (E_c), lattice constants (a, c), and elastic constants (B, C_{ij}) for fcc-Al, hcp-Ti, bcc-Ti, ω–Ti, L1$_2$-Al_3Ti, γ-$Al_{98}Ti_2$, D0$_{22}$-Al_3Ti, L1$_0$-AlTi, hexagonal Ti_3Al, and α-$Ti_{98}Al_2$. The MEAM predictions are in good agreement with experimental and theoretical data, also listed in parentheses.

materials MDPI

Table S3. Cohesive, lattice and elastic constants calculated using the present MEAM potential for the three phases (α, β, and ω) of Ti, the γ-phase of Al, the four most common phases of the intermetallic Al-Ti as well as for Ti and Al substitutions at the dilute limit in γ-Al and α-Ti based alloys. The experimental and theoretical reference values used for the parameterization [7–12] are shown in the parenthesis.

	**α-Ti**	**β-Ti**	**ω-Ti**	**γ-Al**	**γ-$Al_{98}Ti_2$**	**$L1_2$ Al_3Ti**	**$D0_{22}$ Al_3Ti**	**$L1_0$ AlTi**	**Ti_3Al**	**α-$Ti_{98}Al_2$**
E_c	4.840	4.798	4.665	3.357	3.406	3.957	3.944	4.338	4.602	4.803
(eV/at.)	(4.850–4.877)	(4.787–4.792)	(~ α-Ti)	(3.353-3.36)	(~ γ-Al)	(3.950)	(3.920–4.100)	(4.390–4.510)	(4.670–4.780)	(~ α-Ti)
a	2.947	3.274	4.686	4.047	4.034	3.943	3.905	3.921	2.840	2.932
(Å)	(2.92–2.95)	(3.27)	(4.577)	(4.05)	(~ γ-Al)	(3.92)	(3.78–3.96)	(3.95–4.03)	(2.86–2.89)	(~ α-Ti)
c/a	1.580	–	0.601	–	–	–	2.059	1.011	1.606	1.589
	(1.584–1.617)		(0.618)				(2.121–2.251)	(1.014–1.048)	(1.604–1.631)	(~ α-Ti)
B	111	112	106	79	83	116	115	134	131	113
(GPa)	(105–111)	(105–114)	(113)	(79)	(~ γ-Al)	(118)	(106–118)	(112)	(114)	(~ α-Ti)
C_{11}	191	142	184	119	131	228	225	290	320	199
(GPa)	(147–195)	(95–130)	(194–96)	(114)	(~ γ-Al)	(177)	(202–218)	(177–205)	(175–221)	(~ α-Ti)
C_{12}	67	98	104	59	59	60	90	121	44	69
(GPa)	(74–87)	(106–110)	(81–83)	(62)	(~ γ-Al)	(77)	(58–88)	(70–107)	(71–89)	(~ α-Ti)
C_{44}	67	102	15	27	29	57	55	95	66	67
(GPa)	(31–51)	(42–78)	(52–54)	(32)	(~ γ-Al)	(85)	(92–99)	(99–138)	(47–72)	(~ α-Ti)
C_{13}	69	–	36	–	–	–	37	24	38	67
(GPa)	(60–77)		(52–54)				(46–60)	(72–124)	(61–85)	(~ α-Ti)
C_{33}	208	–	234	–	–	–	263	288	294	212
(GPa)	(176–218)		(245)				(218–243)	(171–190)	(219–238)	(~ α-Ti)
C_{66}	–	–	40	–	–	–	146	524	–	–
(GPa)			(56)				(144–145)	(77)		

materials MDPI

We have also tested the reliability of our model with respect to thermal expansion, phonon vibrational spectra, melting points, and phase stability of elemental Al and Ti phases, using the methodology described in Section 2.2 of the manuscript. We find that temperature-dependent equilibrium volumes and lattice thermal expansion of fcc-Al (presented in Figure S4) are in very good agreement with experiments (Ref. [13] and refs therein). The melting point which we estimate, ~850 K, is in reasonable agreement with the experimental value $T_m \approx 935$ K. In addition, the phonon spectra calculated via CMD for fcc-Al at 300 K (see Figure S5) match quite well *ab initio* and experimental results [14,15].

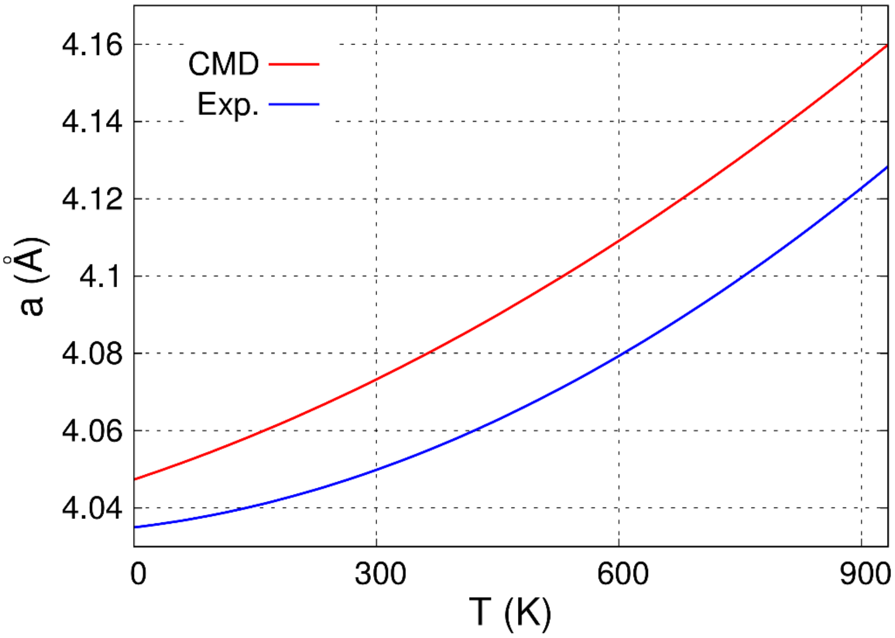

Figure S4. Comparison between CMD and experimental results for the lattice parameter variation with temperature of fcc Al.

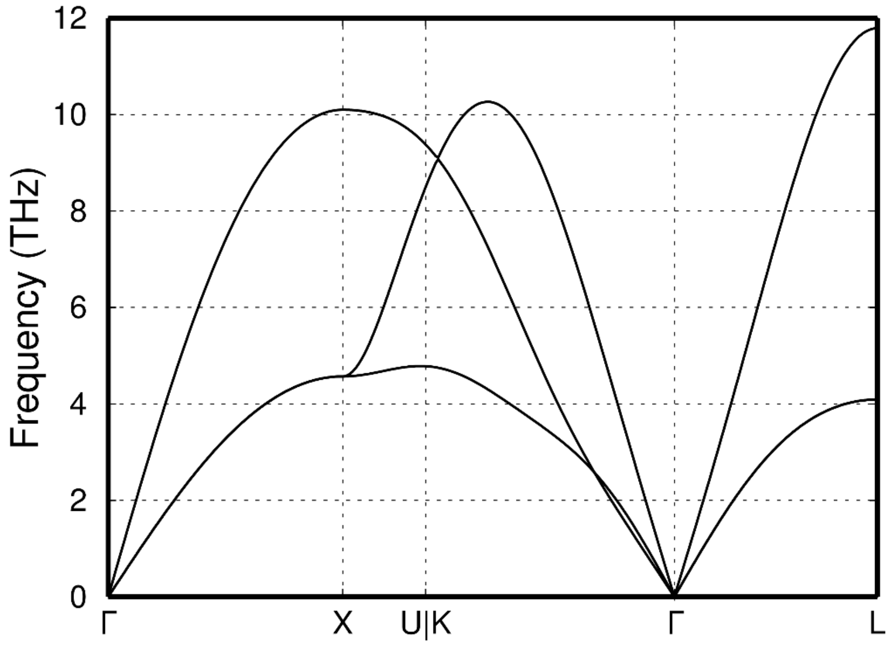

Figure S5. Room-temperature phonon spectra calculated via CMD for fcc-Al.

The equilibrium volume variation with temperature of the hcp- and bcc-Ti phases (Figure S6) are in agreement with experiments (see Ref. [13] and refs. therein), with a maximum discrepancy of 2–3%. Our estimated melting point for pure titanium, ~2500 K, is ~25% larger than the experimental value ~1950 K. The CMD phonon dispersions curves of hcp- and bcc-Ti calculated at room temperature and 1300 K (Figure S7), compare reasonably well with experimental results (see figure 1 in Ref. [16] and figure 2 in Ref. [17]).

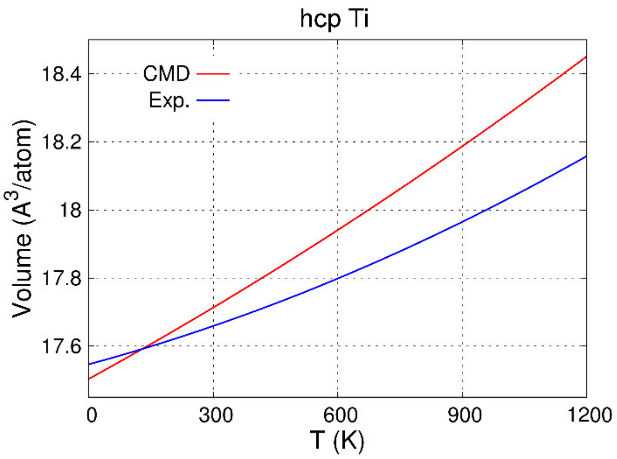

Figure S6. Lattice expansion of Ti (left: hcp, right: bcc structure) in comparison with experiments.

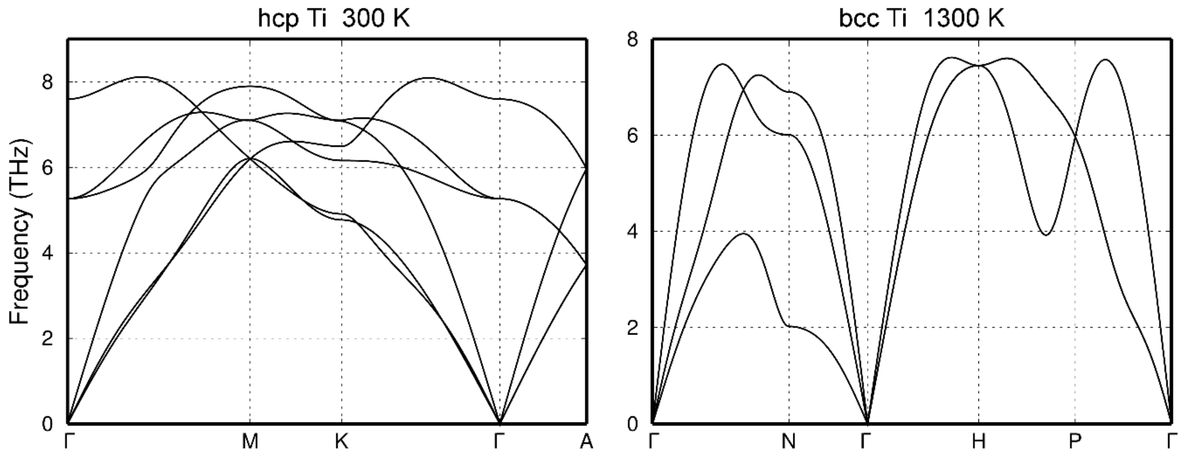

Figure S7. CMD phonon dispersions in Ti; hcp-Ti at 300 K (left), bcc-Ti at 1300 K (right).

Titanium is a particularly difficult system to be modeled using MEAM [18]. This is due to its rich phase diagram and 0 K mechanical instability of the bcc phase, which is stabilized at elevated temperatures by lattice vibrations. It has been shown that modifications to the MEAM formalism are necessary to correctly reproduce its physical properties and phase transitions [7,19]. As a matter of fact, our MEAM parameterization cannot reproduce the hcp- to bcc-Ti phase transition at ~1100 K [20]. The free energy difference $\Delta F_{hcp/bcc}$ calculated at 1200 K, ~48 meV/atom, slowly decreases for $T >$ 1200 K. However, at $T = 1800$ K, the hcp structure is still thermodynamically more stable than the bcc one by ~35 meV/atom. Moreover, our MEAM parameters incorrectly yield a mechanically stable [21] bcc Ti phase at 0 K. In contrast, DFT results yield a C_{12} elastic constant greater than the C_{11} (see table 3 in ref. [7]).

References

1. LAMMPS Molecular Dynamics Simulator. Available online: http.//lammps.sandia.gov/doc/pair_meam.html (accessed on 8 January 2019)
2. Kresse, G.; Hafner, J. Ab initio molecular dynamics for liquid metals. *Phys. Rev. B* **1993**, *47*, 558-561, doi. 10.1103/PhysRevB.47.558.
3. Blöchl, P.E. Projector augmented-wave method. *Phys. Rev. B* **1994**, *50*, 17953-17979, doi: 10.1103/PhysRevB.50.17953.
4. Sangiovanni, D.G.; Alling, B.; Steneteg, P.; Hultman, L.; Abrikosov, I.A. Nitrogen vacancy, self-interstitial diffusion, and Frenkel-pair formation/dissociation in B1 TiN studied by *ab initio* and classical molecular dynamics with optimized potentials. *Phys. Rev. B* **2015**, *91*, 054301, doi: 10.1103/PhysRevB.91.054301.
5. National Institute of Standard and Technology. Available online: https://www.ctcms.nist.gov/potentials/ (accessed on 8 January 2019)
6. OpenKIM – Knowledge of Interatomic Models. Available online: https://openkim.org/ (accessed on 8 January 2019)
7. Hennig, R.G.; Lenosky, T.J.; Trinkle, D.R.; Rudin, S.P.; Wilkins, J.W. Classical potential describes martensitic phase transformations between the alpha, beta, and omega titanium phases. *Phys. Rev. B* **2008**, *78*, 054121, doi: 10.1103/PhysRevB.78.054121.
8. Lee, B.J.; Shim, J.H.; Baskes, M.I. Semiempirical atomic potentials for the fcc metals Cu, Ag, Au, Ni, Pd, Pt, Al, and Pb based on first and second nearest-neighbor modified embedded atom method. *Phys. Rev. B* **2003**, *68*, 144112, doi: 10.1103/PhysRevB.68.144112.
9. Zope, R.R.; Mishin, Y. Interatomic potentials for atomistic simulations of the Ti-Al system. *Phys. Rev. B* **2003**, *68*, 024102, doi: 10.1103/PhysRevB.68.024102.
10. Colinet, C.; Pasturel, A. Ab initio calculation of the formation energies of $L1_2$, $D0_{22}$, $D0_{23}$ and one dimensional long period structures in $TiAl_3$ compound. *Intermetallics* **2002**, *10*, 751-764, doi: 10.1016/S0966-9795(02)00054-7.
11. Materials Project. Available online: https://materialsproject.org/materials/mp-1823/ (accessed on 8 January 2019)
12. Materials Project. Available online: https://materialsproject.org/materials/mp-72/ (accessed on 8 January 2019)
13. Lu, X.G.; Selleby, M.; Sundman, B. Assessments of molar volume and thermal expansion for selected bcc, fcc and hcp metallic elements. *Calphad* **2005**, *29*, 68, doi: 10.1016/j.calphad.2005.05.001.
14. Tang, X.; Li, C.W.; Fultz, B. Anharmonicity-induced phonon broadening in aluminum at high temperatures. *Phys. Rev. B* **2010**, *82*, 184301, doi: 10.1103/PhysRevB.82.184301.
15. Stedman, R.T.; Nilsson, G. Dispersion relations for phonons in aluminum at 80 and 300 K. *Phys. Rev.* **1996**, *145*, 492-500, doi: 10.1103/PhysRev.145.492
16. Stassis, C.; Arch, D.; Harmon, B.N.; Wakabayashi, N. Lattice-dynamics of hcp-Ti. *Phys. Rev. B* **1979**, *19*, 181-188, doi: 10.1103/PhysRevB.19.181.
17. Petry, W.; Heiming, A.; Trampenau, J.; Alba, M.; Herzig, C.; Schober, H.R.; Vogl, G. Phonon dispersion of the bcc phase of Group-IV metals 1. Bcc Ti. *Phys. Rev. B* **1991**, *43*, 10933-10947, doi: 10.1103/PhysRevB.43.10933.
18. Ko, W.-S.; Grabowski, B.; Neugebauer, J. Development and application of a Ni-Ti interatomic potential with high predictive accuracy of the martensitic phase transition. *Phys. Rev. B* **2015**, *92*, 134107, doi: 10.1103/PhysRevB.92.134107.
19. Gibson, J.S.; Srinivasan, S.G.; Baskes, M.I.; Miller, R.E.; Wilson, A.K. A multi-state modified embedded atom method potential for titanium. *Model. Simul. Mater. Sci. Eng.* **2017**, *25*, 015010, doi: 10.1088/1361-651X/25/1/015010.
20. Hellman, O.; Steneteg, P.; Abrikosov, I.A.; Simak, S.I. Temperature dependent effective potential method for accurate free energy calculations of solids. Phys. Rev. B **2013**, *87*, 104111, doi: 10.1103/PhysRevB.87.104111.
21. Mouhat, F.; Coudert, F.X. Necessary and sufficient elastic stability conditions in various crystal systems. *Phys. Rev. B* **2014**, *90*, 224104, doi: 10.1103/PhysRevB.90.224104.

© 2019 by the authors. Submitted for possible open access publication under the terms and conditions of the Creative Commons Attribution (CC BY) license (http://creativecommons.org/licenses/by/4.0/).
